# Anticancer and antioxidant activities of *Pelargonium graveolens* L., *Mentha longifolia* L., and *Chrysanthemum frutescens* L. under salt stress

**DOI:** 10.1038/s41598-026-38277-1

**Published:** 2026-02-20

**Authors:** Asmaa Samy, Nesma M. Helal, Magda M. El-Araby, Walid E. Abdallah

**Affiliations:** 1 Botany Department, Faculty of Science, Ain Shams University, Abbassyia, P.O. 11566, Cairo, Egypt; 2 Chemistry of Medicinal Plants Department, Pharmaceutical and Drug Industries Research Institute, National Research Center, 33 El Buhouth St., Dokki, Giza, 12622 Egypt

**Keywords:** Salinity, *Pelargonium graveolens*, *Mentha longifolia*, *Chrysanthemum frutescens*, Anticancer, HepG2, HCT-116, Physiology, Plant sciences

## Abstract

The goal of the current study was to assess the effects of saline irrigation on the secondary metabolites and essential oil contents of three significant aromatic and medicinal plants, *Pelargonium graveolens* L., *Mentha longifolia* L., and *Chrysanthemum frutescens* L., using different NaCl levels (0, 50, 75, and 100 mM). Our results indicated that the levels of total phenols, flavonoids, tannins, saponins, and alkaloids in the methanolic extracts increased in response to salinity in the three investigated plants. The GC-MS analysis of the essential oils (EOs) showed that some major compounds increased significantly at the high salinity levels (75 and 100 mM NaCl). The EOs anticancer activity of the three plants against the human hepatocellular carcinoma cell line (HepG2) and human colorectal carcinoma (HCT-116) revealed that the *P. graveolens* L. EO exhibited the most potent cytotoxic effect with IC_50_ values of 0.66 and 1.87 µg/ml, at 50 mM NaCl, respectively. The treated *M. longifolia* L. EOs showed the most potent cytotoxic effect against HepG2 and HCT-116 at 75 mM NaCl with IC_50_ values of 2.32 and 7.47 µg/ml, respectively. Otherwise, *M. longifolia* L. crude extracts and EOs exhibited the highest DPPH free radical scavenging activity.

**Supplementary Information:**

The online version contains supplementary material available at 10.1038/s41598-026-38277-1.

## Introduction

Various types of abiotic stresses typically affect plants, such as salinity and drought, which are considered the most severe abiotic stresses that hinder the growth, development, and productivity of crop plants, as well as being the most widespread environmental challenges^1^. Soil salt stress negatively influences biomass, function, metabolism, and enzymatic activities of plants, significantly delaying productivity^2^. The ability of plants to tolerate salinity depends on the interaction between salinity and environmental factors such as soil, water, and climate conditions^3^. Therefore, there are differences in salinity tolerance among species and cultivars, which are influenced by salinity and depend on the severity, duration, and timing of the salt stress^4^.

Secondary metabolites, such as terpenes, phenolic compounds, and alkaloids, are heavily influenced by salinity and serve as adaptive strategies against stress. Additionally, these compounds display antioxidant properties, including the ability to adsorb and neutralize free radicals, scavenge reactive oxygen species (ROS), and decompose peroxide radicals^5–9^. A major group of secondary metabolites is essential oils (EOs), which are complex mixtures of volatile aromatic compounds with relatively low molecular weights, such as carvacrol, thymol, linalool, camphor, 1,8-cineole, menthol, menthone, farnesol, bisabolol, caryophyllene, and others^10^. The yield and composition of EOs are affected by various factors, including salinity levels, seasonal variation, plant growth stage, and the parts of the plant used^11–13^.

Due to their natural ingredients and minimal adverse effects, about 75–80% of people in developing countries have depended on medicinal plants for primary health care for thousands of years worldwide, because they are cheap, available, and have no side effects. Furthermore, 25% of the drugs in the developed countries are herbal drugs. These medicinal plants have benefits for some diseases, such as reproductive system disorders, depression, anxiety disorders, Alzheimer’s disease, and diabetes^14^.

The *Pelargonium* genus contains more than 250 species, one of which is the important aromatic and medicinal plant *Pelargonium graveolens* L., commonly known as rose geranium. It belongs to the Geraniaceae family and is native to South Africa. The main countries producing and exporting geranium oil are China, Egypt, Algeria, and Morocco^15,16^. Commercially, the worldwide cosmetic industry, fragrances, and flavorings utilize the EO of rose-scented geranium. Additionally, it offers several health benefits, including antioxidant, anti-inflammatory, antibacterial, antifungal, antidepressant, and anticancer effects^17^. *P. graveolens* L. EO consists of a mixture of secondary metabolites such as terpenes, esters, aldehydes, ketones, alcohols, and phenols, which help protect the plant from pathogens and herbivores^16^. Research has shown that geranium EO and crude extracts exhibit antioxidant and antimicrobial activities, likely due to their high concentration of volatile compounds and flavonoids, suggesting they could be used as food preservatives^18^. The antitumor properties of *P. graveolens* L. EO have been studied against uterine cervical neoplasia and human promyelocytic leukemia cells^19^. The primary components of geranium EO, β-citronellol and geraniol, can scavenge free radicals, which may play a key role in its antioxidant activity^20^.

*Mentha longifolia* L. is one of the most beneficial medicinal plants with pharmacologically proven natural compounds, commonly known as wild mint or horsemint. This perennial herb is naturally abundant worldwide and belongs to the *Mentha* genus in the Nepetoideae subfamily of Lamiaceae^21,22^. Terpenoids and phenolic compounds represent the conventional secondary metabolites of the Lamiaceae family^12^. Horsemint has a traditional use in treating several diseases, such as gastrointestinal, respiratory, and menstrual disorders, as well as infectious and inflammatory diseases. Additionally, *M. longifolia* L. exhibits several pharmacological activities, such as anti-parasitic, antimicrobial, anti-insect, antimutagenic, antinociceptive, anti-inflammatory, antioxidant, hepatoprotective, anti-diarrhea, and spasmolytic effects^21^.

The Asteraceae family is one of the largest families of higher plants, and their species have been involved in medicine and food for centuries^23^. The *Chrysanthemum* genus is a member of the Asteraceae family, which includes about 300 species possessing biologically active chemical compounds^24^. *Chrysanthemum frutescens* L. (marguerite daisy) has been cultivated for more than 200 years and is native to the Canary Islands^25^. *Chrysanthemum* species possess significant traditional (anti-inflammatory and analgesic effects), pharmacological (anticancer, antimicrobial, immunomodulatory, hepatoprotective, and neuroprotective activities), and industrial (flavor and fragrance) importance because of their secondary metabolites and EOs^26^.

Despite the development of modern medicine, cancer represents one of the biggest challenges in the 21st century. One in six deaths is thought to be because of cancer^27^. Natural products account for approximately 50% of the anticancer drugs used in therapeutic trials during cancer treatment, aiming to minimize side effects due to their multi-targeted properties^28^.

Selecting tolerant plants that still yield economically is one potential strategy to mitigate the negative impacts of salinity. It is essential to utilize these plants in saline land to prevent competition with primary food crops and conserve freshwater used for irrigation^29-31^. Our primary objectives are to utilize therapeutic plants with commercial, pharmacological, and traditional significance that can tolerate salinity, and to investigate how salinity affects the production of secondary metabolites in both the crude extracts and the essential oils of *P. graveolens* L., *M. longifolia* L., and *C. frutescens* L. plants. Additionally, this study illustrates the antioxidant activity of the crude extracts, estimates the free radical scavenging activity of the EOs using the DPPH method, and investigates their antitumor activity against human hepatocellular carcinoma (HepG2) and human colorectal carcinoma (HCT-116) cell lines.

## Materials and methods

### Plant material

We obtained rooted stem cuttings (macro vegetative propagation) of the three studied plant cultivars: *Pelargonium graveolens* L., *Mentha longifolia* L., and *Chrysanthemum frutescens* L., from the farm of Medicinal and Aromatic Plants Research Department, El Kanater El Khairia, Cairo, Egypt.

### Experimental design

We performed the current study in the Botanical Garden of the Botany Department, Faculty of Science, Ain Shams University, during the winter season of 2021–2022, from December to April in *P. graveolens* L., as well as from January to June in the case of *M. longifolia* L. and *C. frutescens* L. The planting of *P. graveolens* L. (30 days old), *M. longifolia* L., and *C. frutescens* L. (20 days old) was in pots 30 × 18 cm filled with 15 kg of clay/sandy (1:2) soil, which were divided into 3 groups; each group represented one plant, which consequently was subdivided into four subgroups. Two months after planting, each subgroup received a particular application of salt concentration as follows: 0 (as control), 50, 75, and 100 mM NaCl, with three replicates of each treatment in a randomized full-block design.

The relative humidity ranged between 30% and 45% minimum and 70% and 90% maximum. Day and night temperatures ranged from 21 to 36 °C and 10–18 °C, respectively. Subsequently, the irrigation of all pots was with tap water or salt to achieve 80% of the filled capacity until the end of the experiment. Finally, after 2 months, we terminated the experiment and the harvest was on April 29 for *P. graveolens* L. and on June 7 for *M. longifolia* L. and *C. frutescens* L. Then the selected plants were collected from each treatment, and the aerial parts were finely powdered after complete drying and kept at 5 °C for carrying out the physiological and biochemical analyses. For GC-MS analysis, the fresh aerial parts were finely kept at 5 °C.

## Methods

### Preparation of methanolic and water extracts

A half-gram weight of air-dried aerial parts from each of the three plants was taken and extracted with either 80% cold methanol (v/v) or distilled water for the following measurements.

### Estimation of total phenols

Total phenols were achieved by the method of^32^, which was estimated by the Folin-Ciocalteu reagent, and the absorbance was measured at 630 nm using a spectrophotometer. The level of total phenols was calculated according to the standard curve of gallic acid and expressed as mg/g dry weight.

### Estimation of flavonoids

The method used in flavonoids estimation was adopted by^33^. The absorbance of the mixture of each extract was measured at 415 nm using a spectrophotometer. The flavonoids content was calculated as mg/g dry weight from a standard curve of quercetin.

### Estimation of tannins

The tannins were evaluated by using saturated sodium carbonate solution and Folin-Ciocalteu reagent as described by^34^. The absorbance of the mixture of each extract was measured at 760 nm using a spectrophotometer. The content of tannins was presented as mg/g dry weight from a standard curve of tannic acid.

### Estimation of saponins

The vanillin reagent was used to calculate the total saponins from the diosgenin standard solution using the^35^ method. The absorbance of methanolic and water extracts was recorded at 560 nm against a blank sample, and then the saponins content was calculated as mg/g dry weight.

### Estimation of alkaloids

The total alkaloids were quantified according to the method described by^33^. The addition of ammonium hydroxide to the two extracts was performed which resulted in the formation of precipitate. The precipitate was dried in the oven until constant dry weights were achieved. The amount of alkaloids in mg/g dry weight was calculated.

### Determination of total antioxidant capacity (TAC)

The total antioxidant capacity of the two extracts was measured by the phosphomolybdenum assay.

Using a spectrophotometer, the absorbance of the reaction was determined at 695 nm. The total antioxidant capacity was expressed as µg/g dry weight from the standard curve of ascorbic acid^36^.

### DPPH radical scavenging activity of plant samples

The antioxidant activity of the extracts of plant samples was estimated using the 1, 1-diphenyl-2-picrylhydrazyl (DPPH) method. Using the following equation, the percentage of DPPH radical scavenging was determined:

$$\% {\text{ DPPH radical scavenging}}={\text{ }}\left[ {\left( {{\mathrm{A}}0{\text{ }}-{\text{ A1 }}} \right)/{\mathrm{A}}0{\text{ }}} \right]{\text{ }} \times {\text{ 1}}00$$

where A0 represents the blank absorbance of the DPPH solution and A1 represents the sample absorbance^37^.

### Extraction and Estimation of the EOs

The 100 g samples of fresh aerial parts of *P. graveolens* L., *M. longifolia* L., and *C. frutescens* L. were steam-distilled for four hours using a Clevenger apparatus to extract the EOs. After collecting the volatile distillate, it was taken with diethyl ether, and dried over sodium sulphate to eliminate any moisture. Then, the ether phase was distilled out over a water bath which kept at 40 °C. For quantitative estimation of EOs, the remaining oils were weighed after evaporation of ether and kept at 5 °C for further analysis^38^.

### GC-MS analysis of the EOs

Gas chromatography-mass spectrometry (GC/MS) (THERMO Scientific Corp., Waltham, MA, USA) with a thermo mass spectrometer detector (ISQ Single Quadrupole Mass Spectrometer; Model ISQ), employing electron ionization (EI) at 70 eV and a spectral range of 40–450 m/z for essential oil analyses in plants. Helium served as the carrier gas in a TR-5 MS column (30 m, 0.32 mm i.d., 0.25 μm), with the injector and detector maintained at 210 °C^39^.

The temperature protocol involved an initial setting of 60 °C for 1 min, followed by a ramp rate of 4.0 °C/min to reach 240 °C, which was then held for an additional minute. Each sample was injected as 1 µL of a 1:10 (v/v) hexane dilution.

### Identification of essential oil constituents

Chemical compounds were identified using Automated Mass Spectral Deconvolution and Identification software (AMDIS)^40^ to compare with mass spectra in the Wiley spectral library collection, GNPS, and NIST library databases (Gaithersburg, MD, USA; Wiley, Hoboken, NJ, USA)^41^. Additionally, to assess their retention indices in relation to n-alkanes (C_6_–C_22_) and mass spectra with those published, or to evaluate the mass spectrum against genuine standards available^42^.

### DPPH radical scavenging activity of the EOs

The antioxidant activity of the EOs was determined at the Regional Center for Mycology and Biotechnology (RCMB) at Al-Azhar University by the DPPH free radical scavenging assay as applied by^43^ in triplicate, and average values were considered. Using the following formula, the DPPH radical’s percentage inhibition (PI) was determined:

1 $${\text{PI }}={\text{ }}\left[ {\left\{ {\left( {{\mathrm{AC}} - {\text{ AT}}} \right)/{\text{ AC}}} \right\}{\text{ x 1}}00} \right]$$

Where *A*C = Absorbance of the control at t = 0 min and *A*T = absorbance of the sample + DPPH at t = 16 min.

The 50% inhibitory concentration (IC_50_), the concentration required for 50% DPPH radical scavenging activity was estimated from graphic plots of the dose-response curve using GraphPad Prism software (San Diego, CA. USA).

### Evaluation of the anticancer activity of the EOs against HepG-2 and HCT-116 cell lines

The Mammalian cell lines HepG-2 (human hepatocellular carcinoma) and HCT-116 (human colon carcinoma) were obtained from VACSERA Tissue Culture Unit.

### Cytotoxicity assay

To conduct the cytotoxicity assay, a 96-well plate containing 1 × 104 cells per well was seeded with 100 µL of growth medium. After that, the EOs were added to three replicates of 96-well plates, resulting in twelve concentrations of each EO. After 24 h, a new medium with various test samples was added. Confluent cell monolayers were dispensed into 96-well flat-bottomed microtiter plates (Falcon, NJ, USA) and two-fold dilutions of the samples were added. For 48 h, the microtiter plates were kept at 37 °C in a humidified incubator with 5% CO_2_. For every test sample concentration, three wells have been set aside. Control cells were cultured with or without DMSO and without test samples. The experiment was found to be unaffected by the small amount of DMSO (maximum 0.1%), which was present in the wells. A colorimetric method^44^ was used to assess the viability of the cells, and^45^ described the process in detail. To determine each tumor cell line’s survival curve following EO treatment, the relationship between surviving cells and EO concentration is plotted. Using GraphPad Prism software (San Diego, CA, USA), the 50% inhibitory concentration (IC_50_) (the concentration needed to cause toxic effects in 50% of intact cells) was estimated from graphic plots of the dose-response curve for each concentration^44^.

### Statistical analysis

The results obtained in this study were expressed as mean values ± standard error (SE). The one-way analysis of variance (ANOVA) test and the least significant difference (LSD) test were used for statistical analysis, with a P value of less than 0.05^46^. Further comparisons were performed between groups, and Duncan’s Multiple Range Test was applied at the 0.05 level. Principal component analysis (PCA) and heatmap were conducted online using the free website https://biit.cs.ut.ee/clustvis.

## Results and discussion

### Phytochemical analysis

In this study, we analyzed the phytochemical constituents of the crude extracts of the air-dried aerial parts of the three investigated plants, *P. graveolens* L., *M. longifolia* L., and *C. frutescens* L., under different levels of salinity (0, 50, 75, 100 mM NaCl). To detect the existence and quantitative estimation of secondary bioactive metabolic compounds in terms of total phenols, flavonoids, tannins, saponins, and alkaloids, we used the methanolic and water extracts. Notably, environmental stresses such as salinity, drought, and heat can improve the assemblage of secondary metabolites in plants^7,47–49^. In this context, our results revealed that the values of total phenols, flavonoids, and tannins exhibited a marked increase in the methanolic extract of the three investigated plants in response to salinity stress compared with their corresponding controls. The percentage of increase in total phenols reached about 123.9%, 101.9%, and 108.7%, respectively, in *P. graveolens* L., *M. longifolia* L., and *C. frutescens* L. plants at 75 mM NaCl in the methanolic extract above the control values (Tables 1, 2 and 3). The apparent increase in flavonoids and tannins content reached 137.3% and 145% in *P. graveolens* L., respectively, at 75 mM NaCl; however, it reached 166.1% and 105% in *M. longifolia* L. at 75 and 50 mM NaCl, respectively. In case of *C. frutescens* L., the increase in flavonoids and tannins were noticeable at 100 mM NaCl level, which reached 116.9% and 114.8%, respectively, in the methanolic extract.

**Table 1:** Effect of salinity stress on total phenols, flavonoids, tannins, saponins, alkaloids, total antioxidant capacity (TAC) contents, and DPPH% on methanolic and water extracts of *P. graveolens* L. plant exposed to salinity for 2 months. Results are shown as a mean of three replicates ± SE. The values with the same letter in the same column are non-significant and with different letters are significant.

Extract type	Salinity levels (mM NaCl)	Total phenols (mg/g dry wt.)	Flavonoids (mg/g dry wt.)	Tannins (mg/g dry wt.)	Saponins (mg/g dry wt.)	Alkaloids (mg/g dry wt.)	TAC (µg/g dry wt.)	DPPH (%)
Methanolic	0	37.6 ± 0.52^c^	25.2 ± 0.44^b^	0.40 ± 0.006^c^	1.62 ± 0.001^ef^	111.5 ± 2.02^a^	4.47 ± 0.01^b^	47.5 ± 0.27^d^
	50	39.8 ± 0.39^c^	25.6 ± 0.36^b^	0.46 ± 0.002^b^	2.42 ± 0.056^c^	88.5 ± 3.75^b^	5.27 ± 0.4^a^	35.4 ± 1.67^e^
	75	46.6 ± 0.75^a^	34.6 ± 2.17^a^	0.58 ± 0.009^a^	1.62 ± 0.001^ef^	116.0 ± 3.46^a^	5.35 ± 0.06^a^	21.1 ± 0.45^f^
	100	43.9 ± 0.94^b^	22.1 ± 0.09^b^	0.45 ± 0.001^b^	1.53 ± 0.003^f^	69.5 ± 2.02^c^	4.81 ± 0.01^ab^	46.6 ± 0.79^d^
Water	0	15.3 ± 0.13^d^	5.94 ± 0.11^cd^	0.11 ± 0.001^ef^	2.15 ± 0.07^cd^	67.5 ± 1.44^c^	2.18 ± 0.05^c^	74.3 ± 0.51^c^
	50	14.7 ± 0.23^d^	6.65 ± 0.06^cd^	0.12 ± 0.001^e^	2.89 ± 0.07^b^	55.0 ± 1.73^d^	2.13 ± 0.06^c^	79.3 ± 0.33^ab^
	75	16.5 ± 0.21^d^	8.82 ± 0.44^c^	0.14 ± 0.001^d^	4.39 ± 0.10^a^	58.0 ± 1.15^cd^	2.72 ± 0.09^c^	77.2 ± 0.19^bc^
	100	11.1 ± 0.03^e^	4.69 ± 0.08^d^	0.09 ± 0.003^f^	1.89 ± 0.02^de^	66.5 ± 2.02^cd^	2.17 ± 0.05^c^	82.2 ± 0.13^a^
LSD at 0.05%		2.58	4.12	0.024	0.304	11.95	0.73	3.79

**Table 2:** Effect of salinity stress on total phenols, flavonoids, tannins, saponins, alkaloids, total antioxidant capacity (TAC) contents, and DPPH% on methanolic and water extracts of *M. longifolia* L. plant exposed to salinity for 2 months. Results are shown as a mean of three replicates ± SE. The values with the same letter in the same column are non-significant and with different letters are significant.

Extract type	Salinity levels (mM NaCl)	Total phenols (mg/g dry wt.)	Flavonoids (mg/g dry wt.)	Tannins (mg/g dry wt.)	Saponins (mg/g dry wt.)	Alkaloids (mg/g dry wt.)	TAC (µg/g dry wt.)	DPPH (%)
Methanolic	0	36.7 ± 0.16^b^	19.76 ± 0.59^b^	0.40 ± 0.008^a^	0.78 ± 0.005^bc^	40.5 ± 2.02^b^	1.72 ± 0.01^b^	78.1 ± 0.11^c^
	50	37.3 ± 0.001^a^	20.18 ± 0.52^b^	0.42 ± 0.005^a^	0.79 ± 0.003^bc^	44.0 ± 2.88^b^	1.76 ± 0.02^b^	76.9 ± 0.23^c^
	75	37.4 ± 0.08^a^	32.83 ± 0.59^a^	0.41 ± 0.002^a^	0.76 ± 0.008^bc^	46.0 ± 6.92^b^	2.24 ± 0.009^a^	76.5 ± 0.21^c^
	100	33.5 ± 0.001^c^	14.78 ± 0.96^c^	0.33 ± 0.001^b^	0.76 ± 0.001^bc^	52.0 ± 4.61^b^	2.02 ± 0.03^ab^	76.9 ± 0.01^c^
Water	0	17.5 ± 0.04^d^	5.23 ± 0.04^d^	0.15 ± 0.010^c^	2.03 ± 0.033^a^	48.5 ± 2.59^b^	2.27 ± 0.19^a^	91.6 ± 0.49^ab^
	50	13.7 ± 0.12^e^	4.31 ± 0.05^d^	0.12 ± 0.003^d^	0.81 ± 0.079^b^	75.0 ± 2.88^a^	2.30 ± 0.007^a^	90.1 ± 1.03^b^
	75	12.5 ± 0.10^f^	4.14 ± 0.03^d^	0.11 ± 0.001^de^	0.85 ± 0.042^b^	74.0 ± 1.15^a^	1.93 ± 0.006^ab^	93.1 ± 0.25^a^
	100	11.9 ± 0.06^g^	3.51 ± 0.01^d^	0.10 ± 0.001^e^	0.56 ± 0.094^c^	75.5 ± 3.17^a^	1.64 ± 0.09^b^	93.4 ± 0.65^a^
LSD at 0.05%		0.47	2.39	0.0215	0.246	18.81	0.4107	2.62

**Table 3:** Effect of salinity stress on total phenols, flavonoids, tannins, saponins, alkaloids, total antioxidant capacity (TAC) contents, and DPPH% on methanolic and water extracts of *C. frutescens* L. plant exposed to salinity for 2 months. Results are shown as a mean of three replicates ± SE. The values with the same letter in the same column are non-significant and with different letters are significant.

Extract type	Salinity levels (mM NaCl)	Total phenols (mg/g dry wt.)	Flavonoids (mg/g dry wt.)	Tannins (mg/g dry wt.)	Saponins (mg/g dry wt.)	Alkaloids (mg/g dry wt.)	TAC (µg/g dry wt.)	DPPH (%)
Methanolic	0	36.5 ± 0.23^b^	20.1 ± 0.59^b^	0.27 ± 0.002^b^	0.61 ± 0.001^b^	42.0 ± 0.001^d^	1.56 ± 0.04^c^	71.8 ± 0.19^b^
	50	39.5 ± 0.09^a^	21.3 ± 0.74^b^	0.28 ± 0.006^b^	0.60 ± 0.006^b^	57.0 ± 0.001^c^	1.60 ± 0.02^c^	62.2 ± 0.01^c^
	75	39.7 ± 0.52^a^	21.9 ± 0.22^ab^	0.29 ± 0.004^b^	0.60 ± 0.007^b^	55.0 ± 0.57^c^	2.18 ± 0.06^b^	56.0 ± 1.41^d^
	100	36.7 ± 0.001^b^	23.5 ± 0.37^a^	0.31 ± 0.011^a^	0.76 ± 0.003^a^	56.5 ± 2.02^c^	2.02 ± 0.05^bc^	62.6 ± 0.03^c^
Water	0	14.4 ± 0.15^c^	6.89 ± 0.07^c^	0.134 ± 0.005^c^	0.50 ± 0.003^c^	82.5 ± 0.86^a^	3.23 ± 0.19^a^	82.6 ± 0.07^a^
	50	13.0 ± 0.14^d^	6.82 ± 0.003^c^	0.114 ± 0.005^cd^	0.42 ± 0.018^d^	83.5 ± 1.44^a^	2.15 ± 0.13^b^	83.1 ± 0.83^a^
	75	13.9 ± 0.15^cd^	7.51 ± 0.18^c^	0.112 ± 0.002^d^	0.14 ± 0.005^e^	68.0 ± 1.15^b^	2.54 ± 0.03^b^	81.6 ± 0.05^a^
	100	13.2 ± 0.35^cd^	6.99 ± 0.06^c^	0.114 ± 0.003^cd^	0.60 ± 0.019^b^	73.5 ± 1.44^b^	2.15 ± 0.13^b^	80.4 ± 0.01^a^
LSD at 0.05%		1.36	2.01	0.021	0.0534	6.11	0.547	3.11

In the water extract, at 75 mM NaCl, the total phenols, flavonoids, and tannins recorded 107.8%, 148.5% and 127.3%, respectively, in *P. graveolens* L., while flavonoids reached 109% in *C. frutescens* L., above the control values. It appears that the increase in production of total phenols and flavonoids, as well as tannins in the three investigated plants, was a part of their response to salinity stress. Our results agree with those observations noticeable by^50–52^, and^7^ who described that salt stress significantly improved phenols and flavonoids in maize, wheat, *Moringa oleifera* Lam., and *Alcea rosea* plants^8^. revealed that total flavonoid and tannin content increased in response to salt in *Origanum majorana*. The most abundant secondary metabolites in plants, phenols and flavonoids, possess crucial antioxidant activities to mitigate the salt stress-triggered ROS overproduction^53^.

In addition, the presented data in Tables (1, 2 & 3) indicated that the salinity levels induced a general increase in saponins and alkaloids in plants under investigation. Saponins showed the most pronounced amount in the methanolic and water extracts of *P. graveolens* L., which increased by approximately 1.5-fold and 2-fold at 50 and 75 mM salinity levels, respectively, compared with the control values. However, the alkaloid content reached about 128.39% and 155.67%, respectively, at 100 mM NaCl in methanolic and water extracts of *M. longifolia* L., above the control values, while the percentage of increase in alkaloid content reached the maximum value at 50 mM NaCl in the *C. frutescens* L. methanolic extract, which recorded 135.7% above the control value. Our findings are in alliance with^54^, who recorded that alkaloid content increased in *Chelidinium majus* L. plant in response to salt stress. The increase in stylopine synthase activity, which is included in alkaloid biosynthesis, might be the reason for the increased alkaloid concentration. Moreover^55] and [56^, reported that higher levels of saponins and alkaloids were noticeable in soybean and milk thistle plants under salt stress.

Populations in developing countries may rely on local traditional medicines as their primary healthcare source^14^. This study showed that in response to salinity, the levels of secondary metabolites (phenols, flavonoids, tannins, saponins, and alkaloids) increased in the aerial parts of the three plants under investigation, which humans can use in traditional medicine. These compounds act as antioxidants, antibacterials, antimicrobials, antivirals, anti-inflammatories, and anti-carcinogens^57–59^.

### Antioxidant and radical-scavenging activities of plant crude extracts of dried aerial parts

The assessment of the antioxidant activity represented that the methanolic and water extracts of the air-dried aerial parts of the three investigated plants exhibited a total antioxidant capacity (TAC) and DPPH free radical scavenging activity. In this study, total antioxidant capacity showed a general increase in methanolic extracts of the three investigated plants exposed to different levels of salt stress (Tables 1, 2 and 3). The percentage of increase reached 119.6%, 130.2%, and 139.7% at 75 mM NaCl, above the control values in *P. graveolens* L., *M. longifolia* L., and *C. frutescens* L., respectively. In the water extracts, our investigation showed that the highest TAC values were at 75 and 50 mM NaCl in *P. graveolens* L. and *M. longifolia* L., respectively.

The antioxidant activity of these plants differs in their DPPH free radical scavenging activity, indicating a degree of nonenzymatic antioxidant effect under stressed and unstressed conditions (Tables 1, 2 and 3). Abushady^60^ stated that the DPPH free radical scavenging capacity showed moderate activity, ranging from 40 to 90%, and remarkable activity when exceeding 90%. The highest values we observed were in the water extracts at 100 mM NaCl in *P. graveolens* L. and *M. longifolia* L. (82.2% and 93.4%, respectively) and at 50 mM NaCl in *C. frutescens* L. (83.1%) compared to the control values. It is worth mentioning that the *M. longifolia* L. methanolic and water extracts recorded the highest DPPH free radical antioxidant activity among the three investigated plants (Tables 1, 2 and 3).

Furthermore, these findings may provide evidence for the physiological role of antioxidant compounds and their action in response to salinity in the three plants, which parallels the variation in total phenolic compounds, flavonoids, tannins, and saponins, as well as alkaloids, across different extracts under salt stress. These extracts comprise a mixture of biologically active compounds, which can act synergistically; therefore, they are involved in the treatment of a wide range of health disorders^58^. Our results revealed that the methanolic and water extracts of the three investigated plants are a powerful source of natural antioxidants. The findings of^61^ support our results, which indicate a positive relationship between phenolic content and antioxidant activity. Similarly, saponins and alkaloids could be involved in the antioxidant biological activity and thereby medicinal uses as reported by^62^.

Plant phenolic compounds exhibit significant antioxidant action, which have been used to treat a number of illnesses. Numerous studies have demonstrated that oxidative stress plays a critical role in the development of neurodegeneration, cancer, cardiovascular complications, muscle degeneration, antibacterial, immune system-promoting, and anti-inflammatory effect^63^. Saponins have several biological effects, including the treatment of venous oedema and male erectile dysfunction, analgesia, anti-nociceptive, antioxidant, antifungal, antidiabetic, and antiplatelet^64^. Alkaloids are involved in the treatment of rheumatoid arthritis due to their immunomodulatory functions; moreover, they possess anticancer properties^62,65^.

**Fig. 1:**
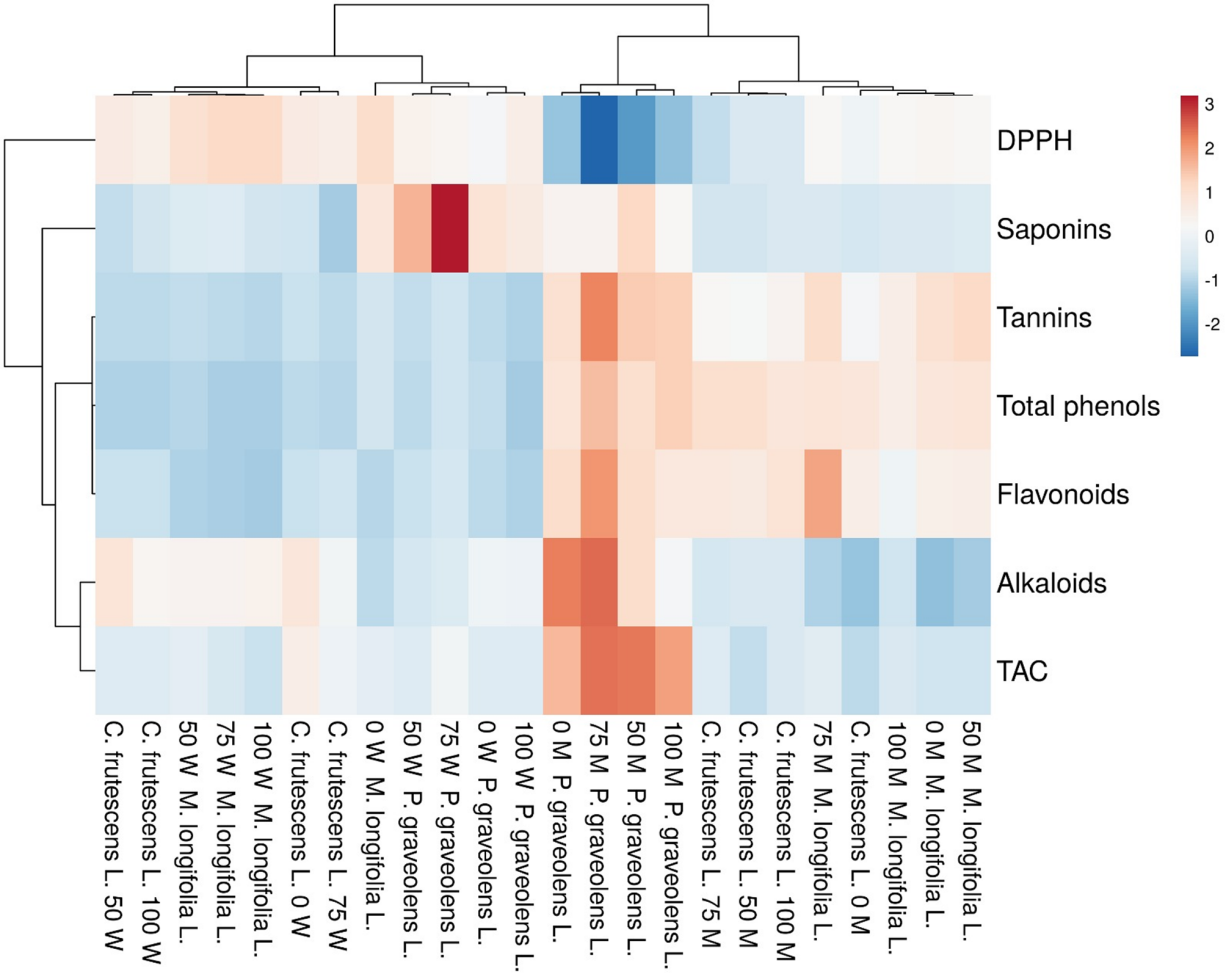
Heatmap showing the correlation between different treatments. A positive correlation between treatments is expressed by the red boxes, while a negative correlation is presented by the blue boxes.

**Fig. 2:**
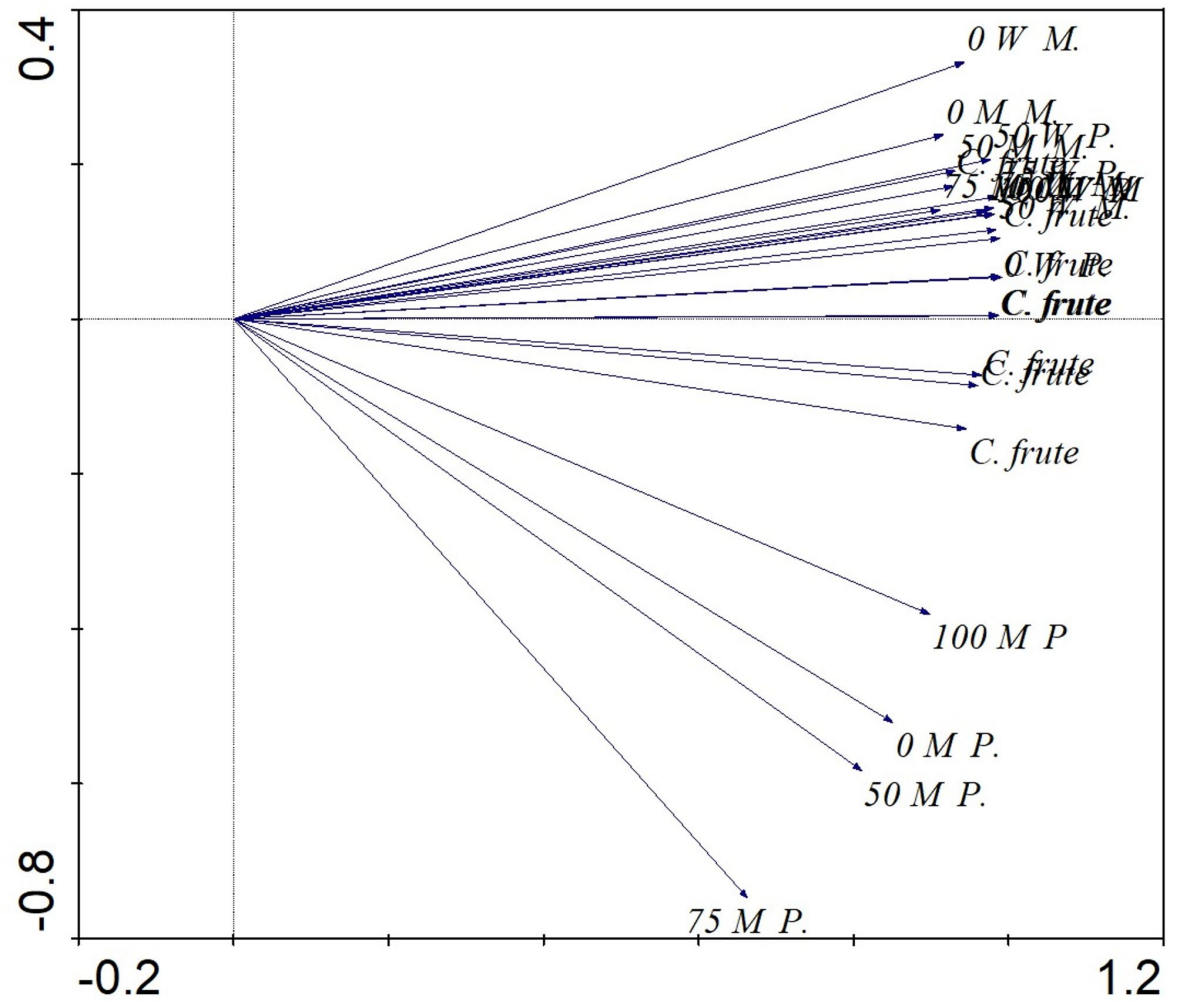
The principal component analysis (PCA), which assembled the similarities between the treatments.

The associations between the variables under investigation were then presented as a cluster heatmap correlation test to provide a summary of the group comparison (Fig. 1). In Fig. 1, the red boxes show a positive correlation between variables, while the blue boxes show a negative correlation. In addition, the principal component analysis (PCA), which compiled the similarities between the samples, was presented by Canoco 4.5 (Fig. 2).

### The EO content and GC-MS analysis

In the current study, the imposition of salt stress showed a non-significant change in yields of the EOs of the three investigated plants (*P. graveolens* L., *M. longifolia* L., and *C. frutescens* L.) with the increase in salinity levels (Table 4). Our results agree with many reports, as^66] and [67^, who reported that the salinity stress non-significantly affected the EO content in *M. longifolia* L. and *P. graveolens* L.

**Table 4:** Effect of salinity on yields of the essential oils of *P. graveolens* L., *M. longifolia* L., and *C. frutescens* L. (ml/100 g dry weight) plants exposed to salinity for 2 months.

Plant name	Salinity levels (mM NaCl)	Yield (ml/100 g dry wt.)
*P. graveolens* L.	0	2.22
	50	2.17
	75	2.158
	100	2.29
*M. longifolia* L.	0	1.611
	50	1.809
	75	1.769
	100	1.516
*C. frutescens* L.	0	0.72
	50	0.718
	75	0.696
	100	0.459

The GC-MS analysis of *P. graveolens* L., *M. longifolia* L., and *C. frutescens* L. EOs are presented in Tables (5, 6 & 7), respectively. In the case of *P. graveolens* L., the GC-MS of the EOs showed the presence of 35 phytoconstituents. α-Citronellol (30.2%), 10-epi-γ-eudesmol (14.01%), geraniol (13.53%), l-menthone (12.32%), linalool (9.87%), geranyl tiglate (2.7%), limonene (2.46%), 2-phenylethyl tiglate (2.06%), germacrene D (1.9%), α-eudesmol (1.49%), Δ-cadinene (1.44%), rose oxide (1.21%), α-bourbonene (1.09%) and geranyl butyrate (1.06%) represent the main constituents of the EO of unstressed *P. graveolens* L. (Table 5). The other constituents were detected in lower amounts than 1%. In addition, it is noticed that the oxygenated monoterpenes comprised the dominant class of the EO (72.42%), followed by oxygenated sesquiterpenes (16.08%), sesquiterpene hydrocarbons (5.49%), monoterpene hydrocarbons (3.61%), and aromatic compounds (2.06%) (Table 6). The findings of this study were in agreement with^18,20,68–70^, who reported that the oxygenated monoterpenes signified the major quantity of the rose-scented geranium EO.

**Table 5:** Effect of salinity on the essential oil chemical compositions of *P. graveolens* L. plant exposed to salinity for 2 months.

Peak No.	RT	Compounds	KI exp.	KI lit.	Rel. %			
					Salinity levels (mM)			
					0	50	75	100
1	6.17	3-Hexen-1-ol	864	853	ND	ND	ND	0.09
2	8.09	(-)-α-Pinene	933	939	0.89	1.24	1.26	1.18
3	10.3	l-Phellandrene	1002	1002	ND	ND	0.13	0.1
4	11.05	Limonene	1020	1029	2.46	2.63	2.66	1.48
5	11.47	β-Ocimene	1030	1037	0.26	0.13	0.45	0.45
6	12.98	cis-Linalool oxide	1067	1072	0.22	0.16	0.22	0.43
7	13.53	Linalool	1080	1094	9.87	9.97	11.11	16.9
8	14.09	Rose oxide	1093	1108	1.21	1.06	2.97	1.51
9	16.73	l-Menthone	1151	1152	12.32	11.22	13.99	13.62
10	17.24	L-(-)-Menthol	1163	1171	0.12	0.1	0.16	0.12
11	17.72	α-Terpineol	1173	1188	0.07	0.1	0.15	0.19
12	19.76	α-Citronellol	1218	1225	30.2	32.09	23.64	25.78
13	20.74	Geraniol	1240	1252	13.53	14.69	9.44	4.72
14	26.3	α-Copaene	1362	1376	0.14	0.15	0.19	0.28
15	27.6	Geranyl acetate	1392	1381	0.06	0.08	ND	ND
16	28.3	trans-Caryophyllene	1407	1419	0.59	0.62	0.97	1.33
17	29.08	Calarene	1426	1433	0.21	0.16	0.23	ND
18	29.9	α-Humulene	1446	1454	0.06	0.04	0.08	0.11
19	30.1	Alloaromadendrene	1450	1460	ND	0.05	0.14	0.17
20	31.42	Germacrene D	1481	1481	1.9	1.94	4.47	4.38
21	31.9	Nerylisobutyrate	1493	1491	0.19	0.19	0.24	0.38
22	32.94	Bicyclogermacrene	1519	1500	0.06	0.08	0.34	0.43
23	33.2	Δ-Cadinene	1525	1523	1.44	1.19	2.03	2.59
24	34.72	Geranyl butyrate	1564	1564	1.06	0.59	0.32	0.46
25	35.1	α-Bourbonene	1574	1569	1.09	0.99	1.42	1.31
26	35.62	(-)-Caryophyllene oxide	1586	1583	0.2	0.11	0.17	0.1
27	35.9	2-Phenylethyl tiglate	1593	1585	2.06	1.54	2.51	3.83
28	36.2	Guaiol	1604	1600	0.1	0.1	0.14	1.99
29	37.42	Geranyl propionate	1634	1643	0.79	0.69	0.92	1.39
30	37.5	10-Epi-γ-eudesmol	1636	1623	14.01	13.2	14.81	9.61
31	38.02	Cubenol	1650	1646	ND	0.07	ND	0.08
32	38.2	α-Eudesmol	1655	1653	1.49	1	1.38	0.77
33	38.31	Alloaromadendrene oxide	1658	1666	0.28	0.16	0.22	0.42
34	38.41	Citronellyl tiglate	1661	1668	0.08	0.1	0.11	0.42
35	39.6	Geranyl tiglate	1693	1696	2.7	3.41	2.82	2.26
		Total %			99.66	99.85	99.69	98.88

RT: retention time index, KI exp.: Kovats retention index reported in the investigation, KI lit.: Kovats retention index from literature, ND: not detected. Rel. %: relative percentage.

**Table 6:** The percentage of compound classes of the essential oils of *P. graveolens* L., *M. longifolia* L., and *C. frutescens* L. under salinity stress.

Plant name	Salinity levels (mM NaCl)	Monoterpene hydrocarbons%	Oxygenated monoterpenes%	Sesquiterpene hydrocarbons%	Oxygenated sesquiterpenes%	Aromatic compounds%	Hydrocarbons%	Oxygenated hydrocarbons%
*P. graveolens*	0	3.61	72.42	5.49	16.08	2.06	ND	ND
	50	4	74.45	5.22	14.64	1.54	ND	ND
	75	4.5	66.09	9.87	16.72	2.51	ND	ND
	100	3.21	68.18	10.6	12.97	3.83	ND	0.09
*M. longifolia*	0	14.78	65.85	3.88	12.47	ND	ND	ND
	50	35.28	45.24	5.86	10.86	ND	ND	ND
	75	43.15	48.92	2.14	5.32	ND	ND	ND
	100	38.84	45.55	3.91	9.01	ND	ND	ND
*C. frutescens*	0	60.27	23.87	4.96	6.34	ND	1.23	0.73
	50	55.06	23.15	7.82	7.14	ND	1.58	2.41
	75	66.39	17.82	4.47	6.07	ND	1.57	1.56
	100	45.42	23.77	8.87	7.73	ND	1.54	2.54

ND: not detected.

The salinity stress induced an increase in the percentage of some main constituents of *P. graveolens* L. EO. The highest percentage of increase of citronellol, geraniol, and geranyl tiglate was noticeable at 50 mM NaCl level, which reached 106.2%, 108.57%, and 126.29%, respectively, above the control values (Table 5). Moreover, oxygenated monoterpenes reached, at the same concentration (50 mM NaCl), about 74.45% compared to the control value (72.42%). At the salinity level of 75 mM NaCl, limonene and 10-epi-γ-eudesmol increased by 108.1% and 105.7% above the control values (Table 5), and monoterpene hydrocarbons showed a significant increase (4.5%) compared with the control value (3.61%) (Table 6). Some major constituents showed a significantly increase at the high salinity levels, 75 and 100 mM NaCl. Linalool (112.5% and 171.2%), rose oxide (245.4% and 124.79%), l-menthone (113.5% and 110.5%), α-bourbonene (130.27% and 120.18%), germacrene D (235.2% and 230.5%), Δ-cadinene (140.9% and 179.8%), and 2-phenylethyl tiglate (121.8% and 185.9%), respectively above the control values (Table 5). These compounds possess anti-inflammatory, antimicrobial, antitumor, antibacterial, and antifungal as well as antioxidant properties^71–77^. Sesquiterpene hydrocarbons recorded a significant increase at the same salinity levels (179.78% and 193.07%, respectively) above the control value (Table 6). Our results are in agreement with those of^78^, which reported that the constituents of volatile oil increased in response to salinity conditions. Figure S1 displays the GC chromatograms of the EOs from *P. graveolens* L. under various salinity levels.

Furthermore, GC-MS analysis indicated that the EO extracts of *M. longifolia* L. have 21 phytoconstituents. The major biologically active components of the untreated *M. longifolia* L. were 1,8-cineole (27.75%), l-menthone (14.68%), α-cadinol (11.26%), sabinene (10.51%), pulegone (8.09%), piperitenone oxide (7.39%), piperitenone (4.46%), carvone (2.82%), trans-caryophyllene (2.68%), and Δ-3-carene (2.49%) as well as α-myrcene (1.6%) (Table 7). The rest of the constituents were found in quantities lower than 1%. Oxygenated monoterpenes (65.85%) represent the dominant class of *M. longifolia* L. EO, followed by monoterpene hydrocarbons, oxygenated sesquiterpenes, and sesquiterpene hydrocarbons (14.78%, 12.47% and 3.88%), respectively (Table 6). The results of this study revealed that the different levels of salinity stress caused a significant increase in some components in *M. longifolia* L. EO. α-Pinene showed an incredibly high rise at all salinity levels, which reached 10.63%, 9.7%, and 20.25%, respectively, at 50, 75, and 100 mM NaCl as compared with the control value (0.18%). In addition, α-myrcene, pulegone, piperitenone, and trans-caryophyllene showed an increase in salinity levels, particularly at 50 mM NaCl that recorded 406.8%, 241.6%, 132.28%, and 146.6%, respectively (Table 7), as well as sesquiterpene hydrocarbons reached 151% at the same level of salinity above the control value (Table 6). At 75 mM NaCl, sabinene and monoterpene hydrocarbons exhibited a highly significant increase, which reached 269.7% and 291.9%, respectively, while Δ-3-carene presented an increase with 100 mM NaCl, which reached 324.89% above the control values (Tables 7 and 6). Various scientific researchers confirmed and supported the antifungal, antibacterial, anti-inflammatory, anticarcinogenic, and antioxidant activities of these compounds^79–85^. Figure S2 displays the GC chromatograms of the EOs from *M. longifolia* L. under various salinity levels.

**Table 7:** Effect of salinity on the essential oil chemical compositions of *M. longifolia* L. plant exposed to salinity for 2 months.

Peak no.	RT	Compounds	KI exp.	KI lit.	Rel. %			
					Salinity levels (mM)			
					0	50	75	100
1	8.04	α-Pinene	931	939	0.18	10.63	9.7	20.25
2	9.41	Sabinene	978	975	10.51	13.19	28.35	10.27
3	10.04	α-Myrcene	995	990	1.6	6.51	5.1	0.23
4	10.4	Δ-3-Carene	1005	1011	2.49	3	ND	8.09
5	11.18	1,8-Cineole	1024	1031	27.75	12.33	20.89	24.36
6	12.3	γ-Terpinene	1051	1059	ND	1.95	ND	ND
7	16.91	l-Menthone	1155	1152	14.68	3.35	11.75	2.23
8	17.81	Isopulegone	1176	1177	0.55	0.31	0.67	ND
9	18.42	Dihydrocarvone 1	1189	1192	ND	ND	ND	0.17
10	20.04	Pulegone	1224	1237	8.09	19.55	14.41	13.5
11	20.31	Carvone	1230	1243	2.82	1.54	ND	0.53
12	21.53	Linalyl acetate	1256	1259	0.11	0.11	ND	0.07
13	25.31	Piperitenone	1340	1343	4.46	5.9	0.61	2.9
14	26.06	Piperitenone oxide	1357	1368	7.39	2.15	0.59	1.79
15	28.61	trans-Caryophyllene	1415	1419	2.68	3.93	1.53	2.86
16	31.6	Germacrene D	1486	1481	0.63	0.98	0.35	0.64
17	33.04	γ-Cadinene	1522	1513	0.57	0.95	0.26	0.41
18	35.05	Caryophyllene oxide	1572	1583	0.4	0.5	0.08	0.16
19	38.13	Cubenol	1653	1646	0.25	0.31	0.08	0.19
20	38.41	α-Cadinol	1661	1654	11.26	9.5	5.03	8.38
21	47.03	Farnesyl acetone	1908	1913	0.56	0.55	0.13	0.28
		Total %			96.98	97.24	99.53	97.31

RT: retention time index, KI exp.: Kovats retention index reported in the investigation, KI lit.: Kovats retention index from literature, ND: not detected. Rel. %: relative percentage.

The GC/MS analysis of *C. frutescens* L. EO identified 35 compounds. The main bioactive components in the untreated plant included cis-ocimene (24.73%), α-myrcene (18.76%), cis-isolimonenol (17.84%), limonene (14.5%), pulegone (4.71%), α-farnesene (3.52%), α-bisabolol (2.42%), 3-tetradecene (1.23%), cubedol (1.16%), and α-pinene (1.29%) (Table 8). The remaining compounds were present at less than 1%. Monoterpene hydrocarbons (60.27%) were the most abundant in untreated *C. frutescens* L., followed by oxygenated monoterpenes, oxygenated sesquiterpenes, and sesquiterpene hydrocarbons (23.87%, 6.34%, and 4.96%, respectively) (Table 6). Our results indicate that the concentration of several EO constituents increased significantly in the treated *C. frutescens* L. plants subjected to different salinity levels. α-Pinene, α-myrcene, and limonene showed maximum increases at 75 mM NaCl, with percentage increases of 410.9%, 168.2%, and 125.9%, respectively, above control values. Similarly, pulegone, α-farnesene, and α-bisabolol increased by 312.1%, 120.1%, and 173.5% at 100 mM NaCl (Table 8). Monoterpene hydrocarbons reached a 110.1% increase at 75 mM NaCl, while sesquiterpene hydrocarbons and oxygenated sesquiterpenes demonstrated notable increases at 100 mM NaCl, with percentage rises of 178.8% and 121.9%, respectively, above control values (Table 6). Several studies have reported the potential roles of these constituents as anti-inflammatories, pesticides, antimicrobials, antifungals, antibacterials, and anticancer agents, as well as for free-radical scavenging activity^80,81,85–88^. Figure S3 displays the GC chromatograms of the EOs from *C. frutescens* L. under various salinity conditions.

**Table 8:** Effect of salinity on the essential oil chemical compositions of *C. frutescens* L. plant exposed to salinity for 2 months.

Peak no.	RT	Compounds	KI exp.	KI lit.	Rel. %			
					Salinity levels (mM)			
					0	50	75	100
1	8.09	α-Pinene	933	939	1.29	3.42	5.3	3.24
2	9.56	Artemiseole	980	976	0.29	0.24	0.09	ND
3	10.01	α-Myrcene	994	990	18.76	25.5	31.56	16.42
4	11.05	Limonene	1020	1029	14.5	13.77	18.25	12.03
5	11.55	cis-Ocimene	1032	1037	24.73	11.8	10.27	13.15
6	15.39	Alloocimene	1122	1132	0.99	0.57	1.01	0.58
7	15.48	cis-Isolimonenol	1124	1133	17.84	11.15	10.9	7.21
8	16.8	trans-Limonen oxide	1153	1142	0.12	0.13	0.06	0.37
9	17.01	Menthone	1158	1152	0.57	1.05	0.74	1.22
10	20.1	Pulegone	1226	1237	4.71	10.42	5.82	14.7
11	24.93	trans-Carvyl acetate	1332	1342	0.34	0.16	0.21	0.27
12	25.43	α-Cubebene	1343	1351	ND	0.33	0.06	ND
13	26.51	α-Copaene	1367	1376	0.22	0.18	0.23	0.41
14	27.22	3-Tetradecene	1383	1387	1.23	1.58	1.57	1.54
15	28.13	α-Cedrene	1404	1411	ND	0.61	ND	ND
16	28.91	trans-Caryophyllene	1422	1419	0.11	1.1	0.1	0.21
17	30.51	α-Humulene	1460	1454	ND	ND	ND	1.26
18	32.01	Germacrene D	1495	1481	0.97	0.26	0.24	0.65
19	32.52	α-Bisabolene	1509	1507	0.14	0.12	0.08	ND
20	32.84	α-Farnesene	1517	1505	3.52	3.9	3.45	4.23
21	33.2	trans-Calamenene	1526	1522	ND	1.32	ND	1.45
22	33.49	Δ-Cadinene	1533	1523	ND	ND	0.31	0.66
23	33.81	Nerolidol	1541	1532	0.66	0.11	0.12	ND
24	35.43	Caryophyllene oxide	1582	1583	0.07	0.19	0.23	0.23
25	35.94	Cubedol	1595	1580	1.16	1.04	0.78	0.29
26	37.54	Gossonorol	1638	1637	0.33	0.71	0.44	0.8
27	37.73	Torreyol	1643	1646	0.1	0.12	0.08	0.19
28	37.81	tau-Muurolol	1645	4642	0.2	0.34	0.22	0.41
29	37.96	α-Cadinol	1649	1654	ND	0.4	ND	0.49
30	39.02	α-Bisabolol	1677	1685	2.42	4.02	2.32	4.2
31	40.82	Farnesol	1727	1723	0.51	0.13	ND	1.12
32	41.53	α-Sinensal	1747	1756	ND	0.08	0.99	ND
33	41.91	cis-Lanceol	1758	1761	0.89	ND	0.89	ND
34	45.63	Hexadecanol	1866	1875	0.73	1.32	0.92	2.54
35	50.91	Falcarinol	2031	2036	ND	1.09	0.64	ND
		Total %			97.4	97.16	97.88	89.87

RT: retention time index, KI exp.: Kovats retention index reported in the investigation, KI lit.: Kovats retention index from literature, ND: not detected. Rel. %: relative percentage.

### Anticancer activity of the EOs

To examine the anticancer effects of the EOs from *P. graveolens* L., *M. longifolia* L., and *C. frutescens* L., experiments were performed using cultured human hepatocellular carcinoma (HepG2) and human colorectal carcinoma (HCT-116) cell lines. The results in this study indicated that the incubation of HepG2 and HCT-116 cell lines with the three investigated EOs for 24 h reduced the viability of the two cell lines, resulting in cytotoxic effects ranging from high to moderate activity. A compound is considered to have high cytotoxic activity if the IC_50_ value is less than 20 µg/ml, according to the US National Cancer Institute (NCI). The IC_50_ values correspond to three other levels of cytotoxicity: moderate (21–200 µg/ml), weak (201–500 µg/ml), and nonexistent (˃ 500 µg/ml)^89^.

The *P. graveolens* L. EO exhibited a potent cytotoxic activity, which increased with increasing salt concentration. The more pronounced IC_50_ values of *P. graveolens* L. against HepG2 cell lines were noticeable at 50 mM salinity level, followed by 100 mM NaCl level, which recorded 0.66 and 1.06 µg/ml, respectively (Table 9 & S1 and Figure S4). Moreover, the IC_50_ values of HCT-116 cell lines exhibited 1.87 and 3.82 µg/ml, respectively, at the same salinity levels (Table 9 & S2 and Figure S5). Previous studies revealed that *P. graveolens* L. EO performed effective anticancer activity against human promyelocytic leukemia and uterine cervical neoplasia^90^. Monoterpenes class in terms of hydrocarbons and, oxygenated are the major constituents represented in the EO of *P. graveolens* L., which reached 78.45% at 50 mM NaCl level as compared to the control value (76.03%) (Table 6), as well as the main constituents, citronellol, geraniol, and geranyl tiglate (Table 5). The results in this investigation are in agreement with the assumption that the EOs with high concentrations of monoterpenes are documented as food preservers, which are considered natural antioxidants and are also active against mammary, lung, skin, liver, and forestomach cancers. Certainly, monoterpenes have antitumor activity, inhibiting the formation and growth of cancer, which induces tumor regression. Geraniol enhanced the 5-fluorouracil treatment of human colon cancer cells and reduced the growth of leukemia, melanoma, hepatoma, and pancreatic cancer cells. Citronellol is an oil-soluble constituent derivative from geranium that exhibits antitumor activity against breast and lung cancers, causing apoptosis and necroptosis, as well as having anti-inflammatory effects^19,90–92^. Linalool possesses the ability to trigger apoptosis of cancer cells thus, it used as anticancer substance^73^. The antioxidant and anticancer activities of the geranium EO’s main constituents may be responsible for its strong cytotoxic effect by acting synergistically with the other constituents.

**Table 9:** Antitumor activity of the essential oils of *P. graveolens* L., *M. longifolia* L., and *C. frutescens* L. plants exposed to salinity for 2 months against human hepatocellular cancer cell line (HepG2) and human colorectal carcinoma (HCT-116) (IC_50_ µg/ml).

Plant name	Salinity levels (mM NaCl)	HepG2 (IC_50_ µg/ml)	HCT-116 (IC_50_ µg/ml)
*P. graveolens*	0	8.94 ± 0.63	23.35 ± 0.91
	50	0.66 ± 0.14	1.87 ± 0.23
	75	2.59 ± 0.51	8.75 ± 0.75
	100	1.06 ± 0.27	3.82 ± 0.34
*M. longifolia*	0	3.37 ± 0.65	4.47 ± 0.69
	50	13.13 ± 0.86	28.96 ± 1.28
	75	2.32 ± 0.47	7. 47 ± 0.62
	100	25.81 ± 0.94	39.42 ± 2.17
*C. frutescens*	0	42.07 ± 1.85	58.76 ± 1.08
	50	47.55 ± 1.96	62.21 ± 1.23
	75	105.23 ± 2.84	175.78 ± 6.52
	100	77.51 ± 2.17	122.91 ± 3.44
Control	Doxorubicin	2.62 ± 0.09	0.49 ± 0.07

The treated *M. longifolia* L. EOs showed a potent cytotoxic effect against HepG2 with IC_50_ values of (13.13 and 2.32 µg/ml) at 50 and 75 mM NaCl, respectively (Table 9 & S3 and Figure S6). On the other hand, the potent cytotoxic effect for HCT-116 was at 75 mM NaCl with IC_50_ 7.47 µg/ml (Table 9 & S4 and Figure S7). Menthone (11.75%), one of the main constituents in the EO of *M. longifolia* L. (Table 7), exhibits a range of biological activity, including antibacterial, antifungal, anticancer, and anti-inflammatory properties^77,93^. Mamur^93^ revealed that menthone significantly reduced the cell viability (%) after 24 h against the human breast cancer (MCF-7) cell line. Moreover, previous studies demonstrated that α-pinene induces cell cycle arrest, which has antitumor activities^94^, as well as 1,8-cineole induces apoptosis to inhibit the proliferation of colon cancer cells^95^. Myrcene’s cytotoxic action targets a wide range of cancer cells by inhibiting their proliferation, such as P388 leukemia cells, HT-29 colon adenocarcinoma, MCF-7 breast cancer, lung cancer cells, and different tumor cell lines^85^. Moreover, α-cadinol, sabinene, and pulegone possess an antioxidant effect^81,96–98^.

Moderate cytotoxic effects of *C. frutescens* L. EOs in the HepG2 and HCT-116 cell lines have been observed. The noticeable activity was detected at 50 mM NaCl (IC_50_ 47.55 and 62.21 µg/ml, respectively) for HepG2 and HCT-116 compared to the other salinity levels (Table 9, S5 & S6 and Figures S8 & S9). At the same salinity level, α-pinene, α-myrcene, pulegone, α-farnesene, and α-bisabolol showed a detectable increase in response to salinity. Limonene possesses anticancer properties, particularly concerning liver and stomach cancer, as well as in transdermal application, so it is used as an additive to increase the penetration of active substances^99^. The effects of limonene have been investigated on human bladder cancer cells (T24), leading to cell cycle arrest, suppression of cell migration, invasion, and apoptosis^92^. Moreover, one common sesquiterpene alcohol with antigliomale properties is α-bisabolol^100^. Myrcene has a cytotoxic effect against many different cancer cells, including P388 leukemia cells, HT-29 colon adenocarcinoma, MCF-7 breast cancer, lung cancer cells, and various tumor cell lines due to inhibition of proliferation^85^. It is also necessary to consider the synergistic effects of these active chemicals with the other constituents of the EO, regarded as cytotoxic effects of the EO of *C. frutescens* L. The presence of extremely hydrophobic, low molecular weight components was responsible for the increase in the cytotoxicity of the EOs of the three plants. These substances have the potential to readily cross the membrane and/or interact with it, resulting in a loss of structural integrity; consequently, the increased susceptibility of protons and ions may result in cell death^101^.

### DPPH free radical scavenging activity of the EOs

The DPPH free radical scavenging activity of *P. graveolens* L., *M. longifolia* L., and *C. frutescens* L. EOs at different salinity levels, expressed as IC_50_, was determined and calibrated with that of the standard antioxidant ascorbic acid. The antioxidant activity of the EO of *P. graveolens* L. increased with the increase in salinity levels compared with the control value. The more pronounced DPPH IC_50_ value of *P. graveolens* L. EO was observed at 50 mM NaCl, which recorded 30.62 µg/ml, followed by 75 and 100 mM NaCl with IC_50_ values of 37.61 and 45.95 µg/ml, respectively as compared to control value, 81.59 µg/ml (Table 10). This indicated that, there are a positive correlation between the antioxidant and anticancer activity of *P. graveolens* L. EO against both investigated human HepG2 and HCT-116 cell lines. Fayed^68^ confirmed our results, who concluded that the antioxidant and anticancer activities of geranium EO were more potent than those obtained from the EO of *Petitgrain mandarin.*

**Table 10:** The essential oils DPPH free radical scavenging activities of *P. graveolens* L., *M. longifolia* L., and *C. frutescens* L. plants exposed to salinity for 2 months (IC_50_ µg/ml).

Plant name	Salinity levels (mM NaCl)	DPPH (IC_50_ µg/ml)
*P. graveolens*	0	81.59 ± 3.62
	50	30.62 ± 1.29
	75	37.61 ± 2.74
	100	45.95 ± 2.65
*M. longifolia*	0	11.09 ± 0.59
	50	12.18 ± 0.63
	75	14.08 ± 0.43
	100	9.80 ± 0.29
*C. frutescens*	0	709.44 ± 16.89
	50	465.12 ± 11.08
	75	261.62 ± 9.71
	100	376.86 ± 10.68
Control	Ascorbic acid	10.21 ± 0.77

*M. longifolia* L. EOs showed the most potent antioxidant activity of the three investigated plants compared with ascorbic acid (Table 10). The highest antioxidant activity was observed at 100 mM NaCl level with an IC_50_ value of 9.8 µg/ml compared to the control value (11.09 µg/ml). Pulegone, a well-known substance belonging to the Lamiaceae family, has strong antioxidant properties^94^. The antioxidant scavenging activity of DPPH was significantly observed in the EO of *M. longifolia* L. in the study carried out by^102^.

Further, the DPPH IC_50_ values of *C. frutescens* L. EOs decreased with all salinity levels, particularly at the 75 mM NaCl level, which measured an IC_50_ value of 261.62 µg/ml as compared with the control value (709.44 µg/ml) (Table 10). α-Pinene, α-myrcene, and limonene reached their highest concentrations at the same level of salinity, which may participate in the enhancement of the EOs’ antioxidant activity. The EOs of these three plants are promising sources of antioxidant compounds that have been used in traditional medicine and as food preservatives^19,25,102^.

According to our results, we can illustrate that *P. graveolens* L., *M. longifolia* L. and *C. frutescens* L. plants can withstand and resist salinity parallels with the enhancement of their antioxidant and antitumor activities. Among the three investigated plants, *P. graveolens* L. methanolic extract showed the highest TAC. Additionally, *P. graveolens* L. and *M. longifolia* L. EOs performed the most potent antitumor activity, while *M. longifolia* L. crude extracts and EOs recorded the highest DPPH free radical antioxidant activity.

This study found that the water extracts and essential oils of the three aromatic plants exhibit DPPH free-radical-scavenging activity across different salinity levels, which have been used to treat several illnesses associated with oxidative stress. Consequently, this supports their use as traditional medicines. Moreover, since these aromatic plants are important sources of EOs, which are promising for anticancer treatment, they are used in the pharmaceutical industry.

## Conclusion

In this connection, different salinity levels caused an increase in phenolics, flavonoids, tannins, saponins, and alkaloids in the methanolic extract of the three investigated plants. *M. longifolia* L. crude extracts and EOs recorded the highest DPPH free radical antioxidant activity in response to salinity. Moreover, some main constituents increased in the EOs at different levels of salinity, such as geraniol, citronellol, linalool, limonene, menthone, rose oxide, 10-epi-γ-eudesmol, geranyl tiglate, 2-phenylethyl tiglate, germacrene D, Δ-cadinene, and α-bourbonene in *P. graveolens* L. On the other hand, α-pinene, α-myrcene, and pulegone increased in the EOs of *M. longifolia* L. and *C. frutescens* L. Furthermore, sabinene, piperitenone, Δ-3-carene, and trans-caryophyllene increased in *M. longifolia* L. Moreover, limonene, α-farnesene, and α-bisabolol increased in *C. frutescens* L. EO. Furthermore, the *P. graveolens* L. and *M. longifolia* L. EOs are more effective against HepG2 and HCT-116 cell lines, followed by *C. frutescens* L. Therefore, we have beneficial suggestions for further studies on the natural extraction and separation of these components or using a mixture of these biologically active constituents to enhance their biological and antioxidant activities. Moreover, further biological studies are required for the crude extracts and the EOs of the three aromatic plants, such as anticancer activity (against other cell lines), anti-inflammatory effect, and antimicrobial activities. Furthermore, investigating their toxicity on the non-cancerous human cell line (healthy cells) is also a priority for future investigations.

## Supplementary Information

Below is the link to the electronic supplementary material.

Supplementary Material 1

## Data Availability

The authors declare that all data generated or analyzed during this investigation are involved in this published article.
