# Supplementary material for: Anticancer and antioxidant activities of Pelargonium graveolens L., Mentha longifolia L., and Chrysanthemum frutescens L. under salt stress
Source: Sci Rep. 2026 Feb 20;16:7478. doi: 10.1038/s41598-026-38277-1 (PMC12929598; doi:10.1038/s41598-026-38277-1)
Supplement: Supplementary file 1 — Supplementary Material 1 [file 41598_2026_38277_MOESM1_ESM.docx]

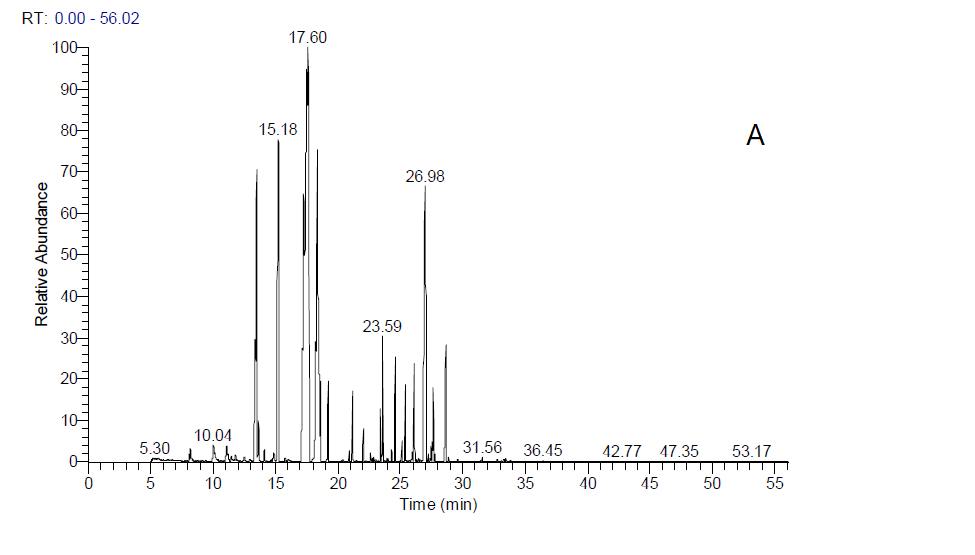


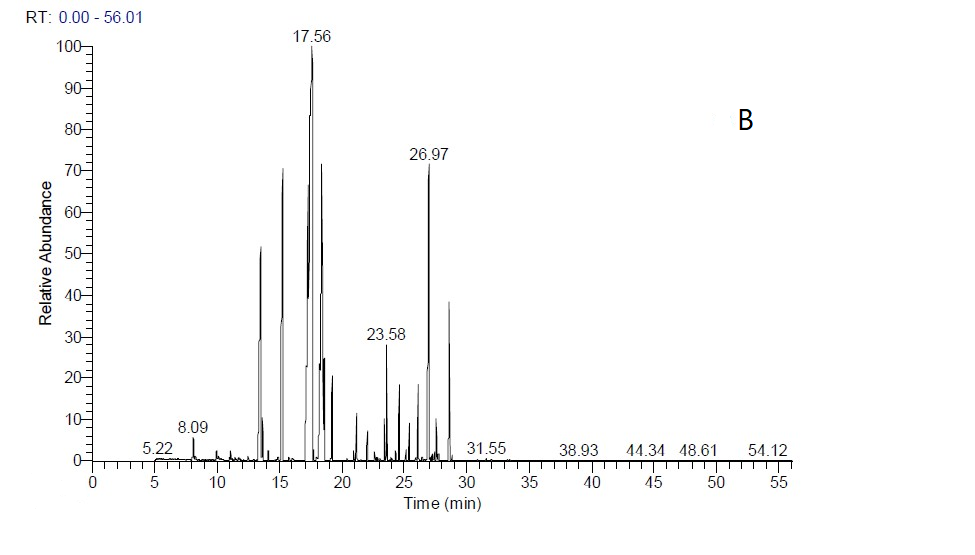


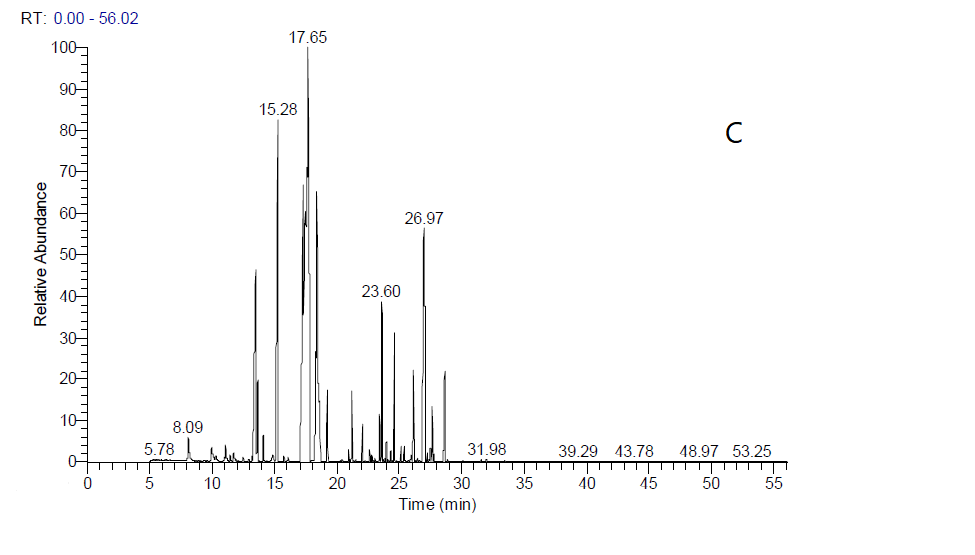


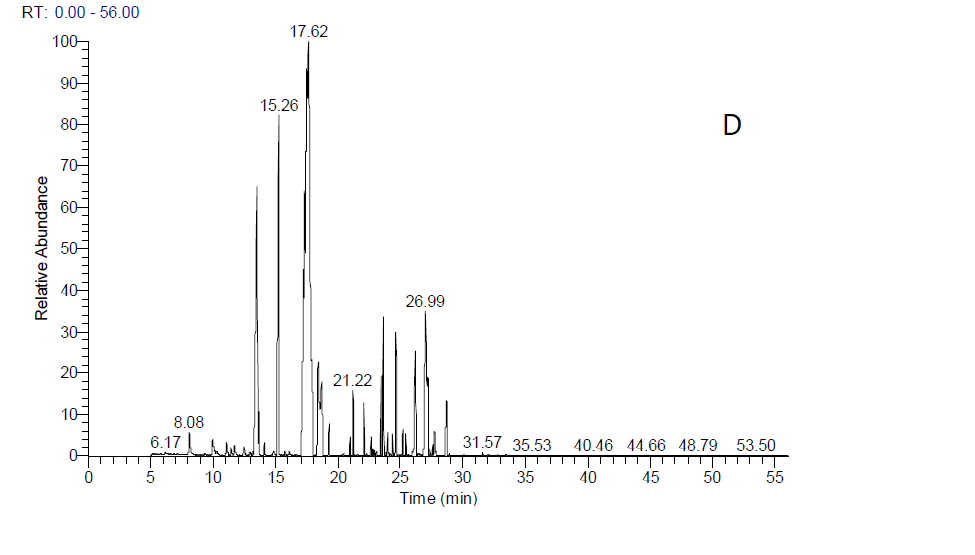


**Fig S1.** GC Chromatograms of the EOs of *P. graveolens* L. under salinity stress. A, B, C, and D represent 0, 50, 75, and 100 mM NaCl respectively.

**
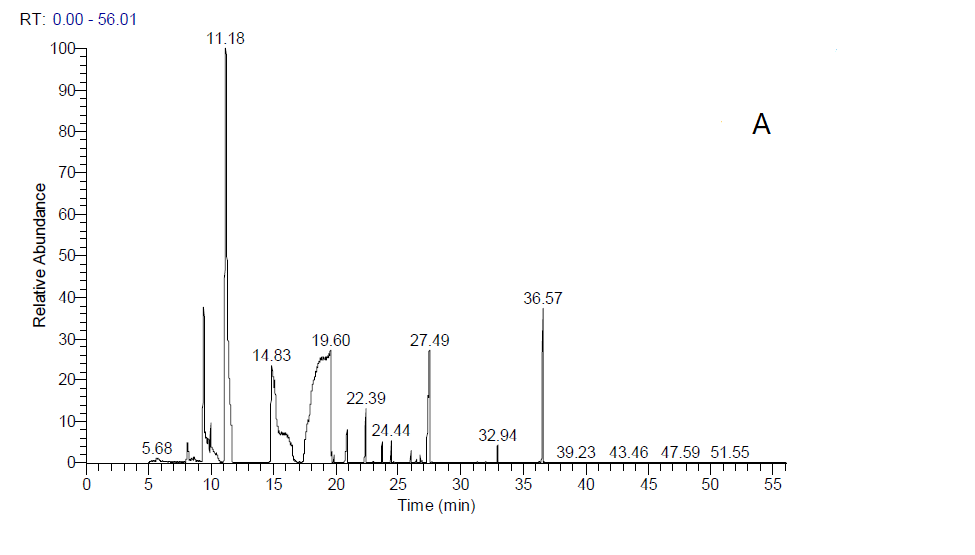
**

**
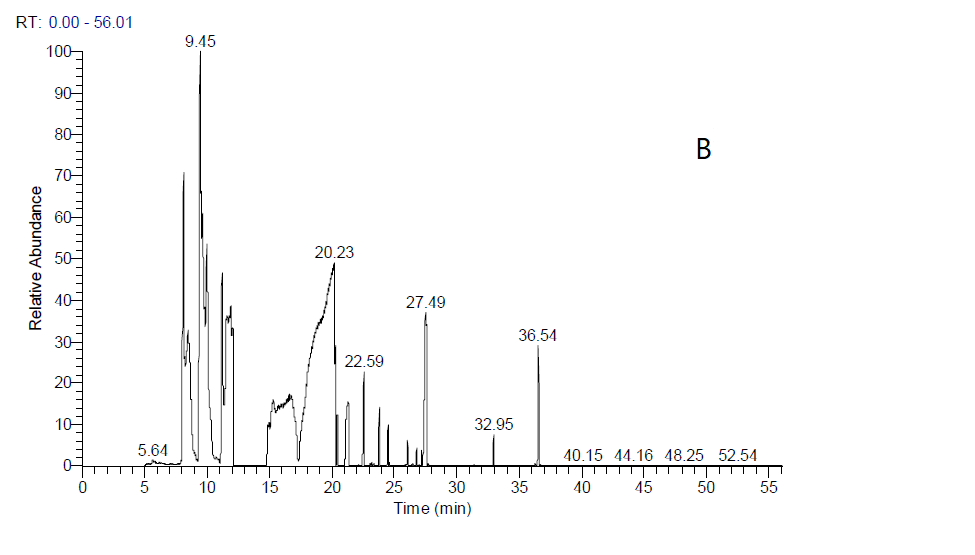
**

**
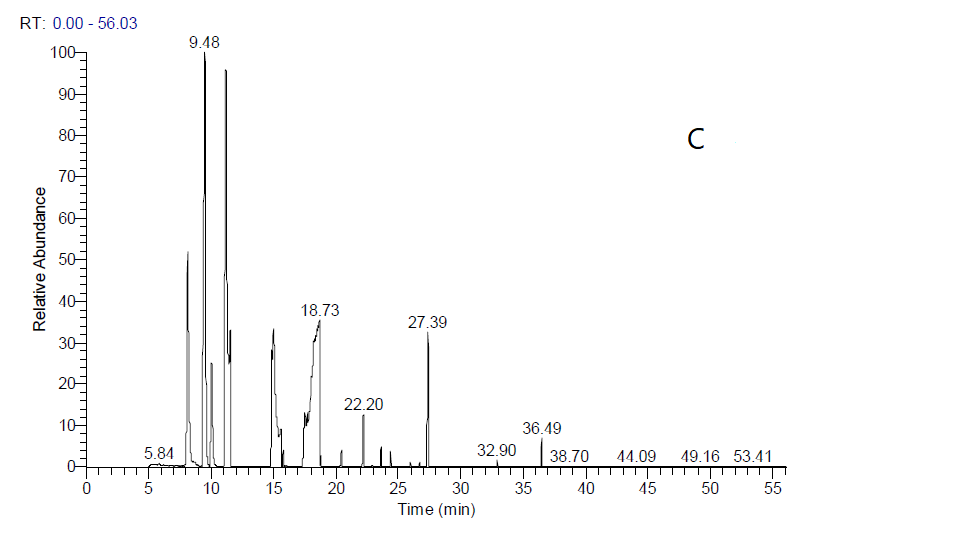
**


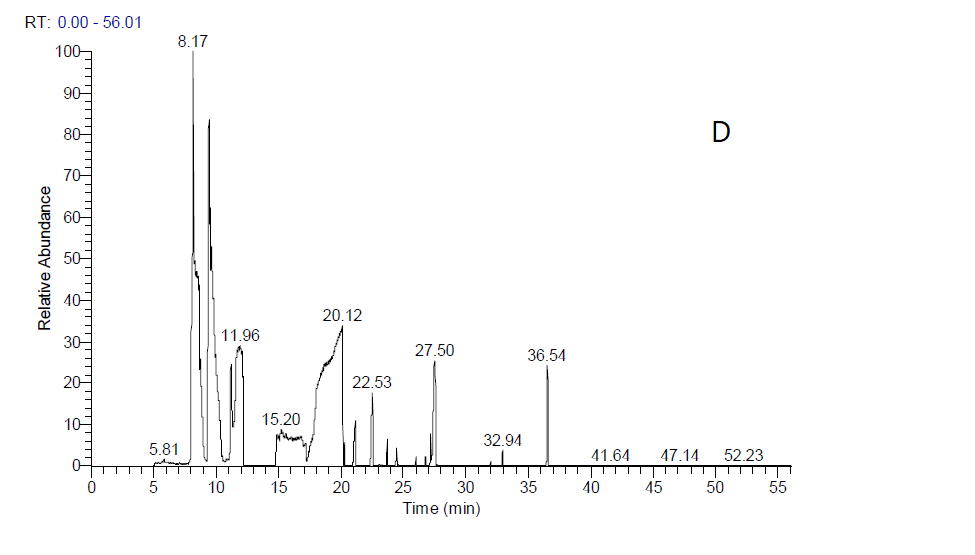


**Fig S2.** GC Chromatograms of the EOs of *M. longifolia* L. plant under salinity stress. A, B, C, and D represent 0, 50, 75, and 100 mM NaCl respectively

**
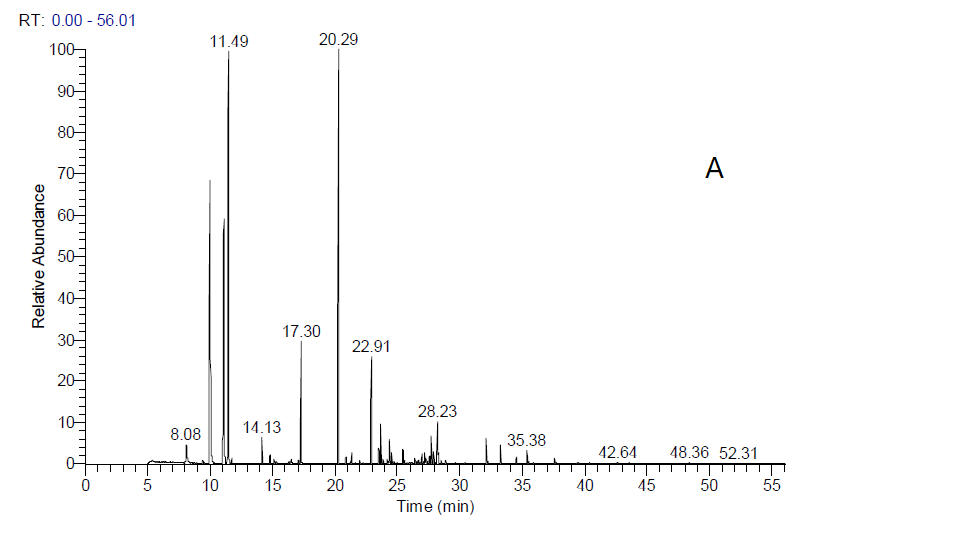
**

**
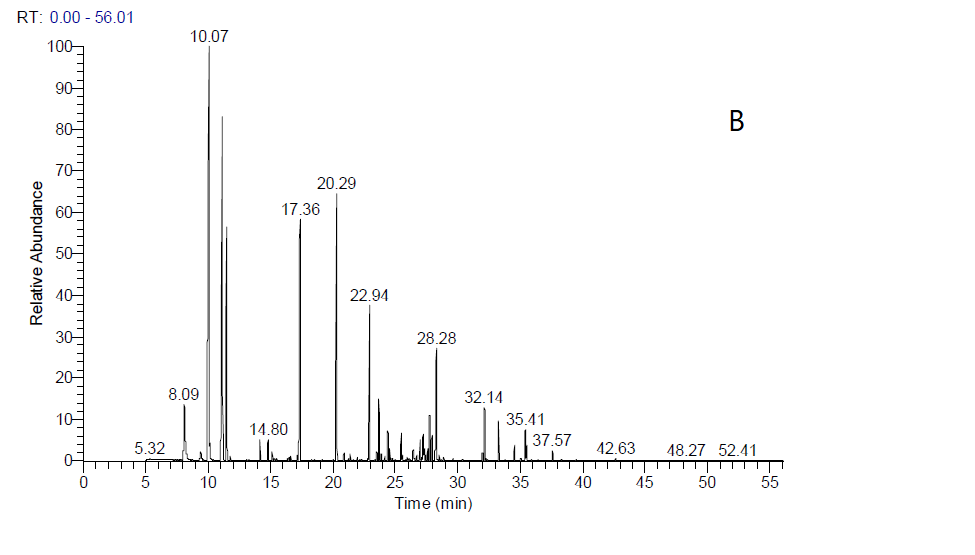
**


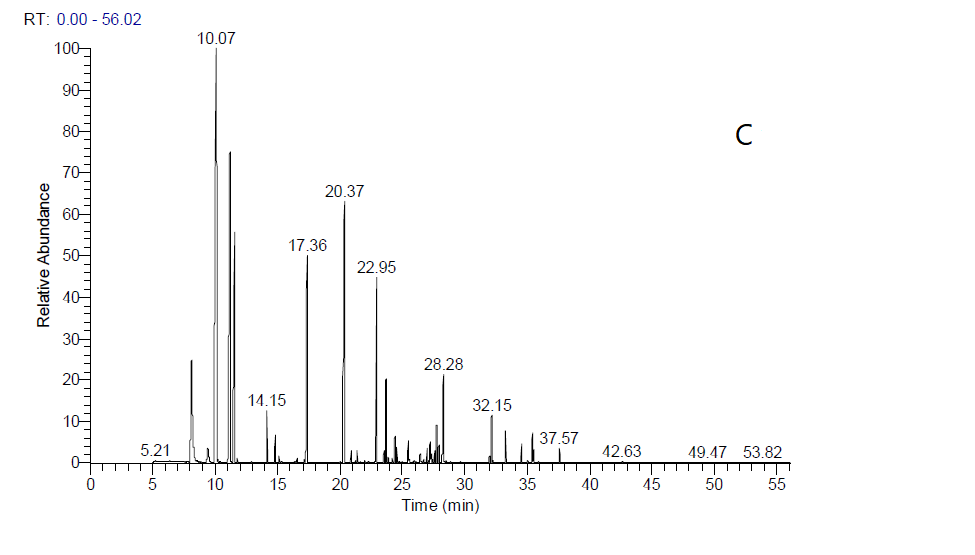


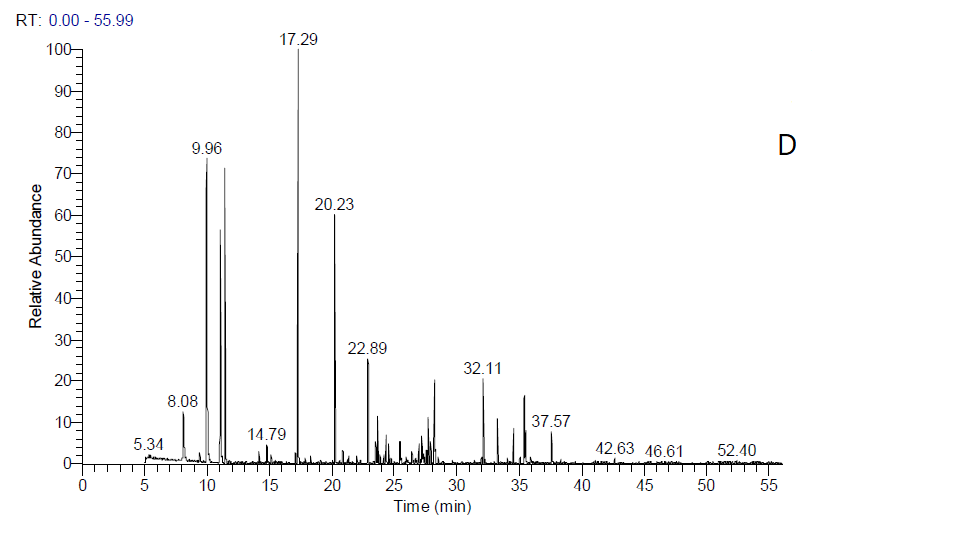


**Fig S3.** GC Chromatograms of the EOs of *C. frutescens* L. plant under salinity stress. A, B, C, and D represent 0, 50, 75, and 100 mM NaCl respectively


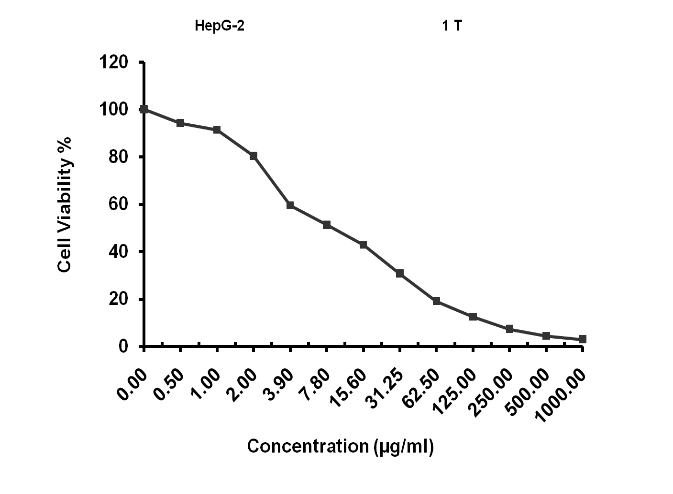

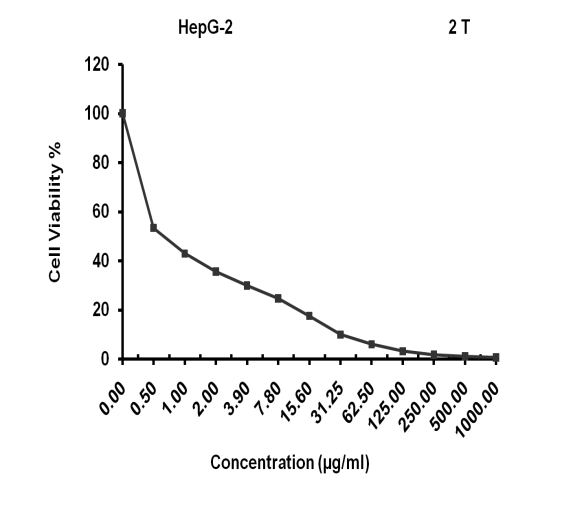


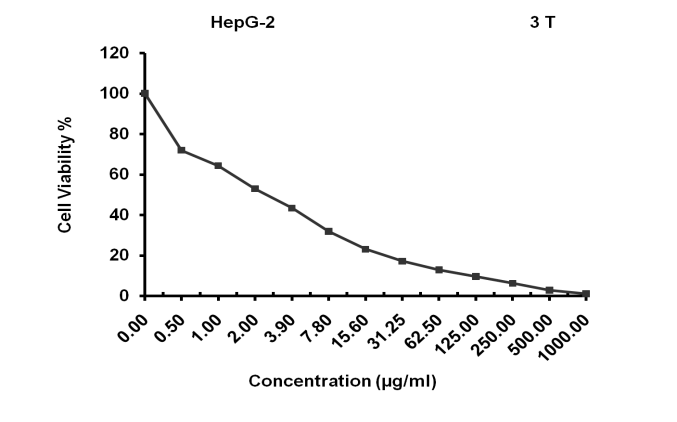

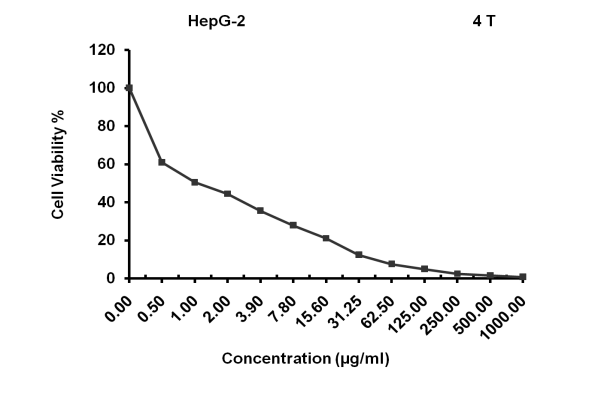


**Fig S4.** Inhibition of HepG-2 cells by the essential oil of *P. graveolens* L. 1T, 2T, 3T and 4T represent 0, 50 75 and 100 mM NaCl, respectively.


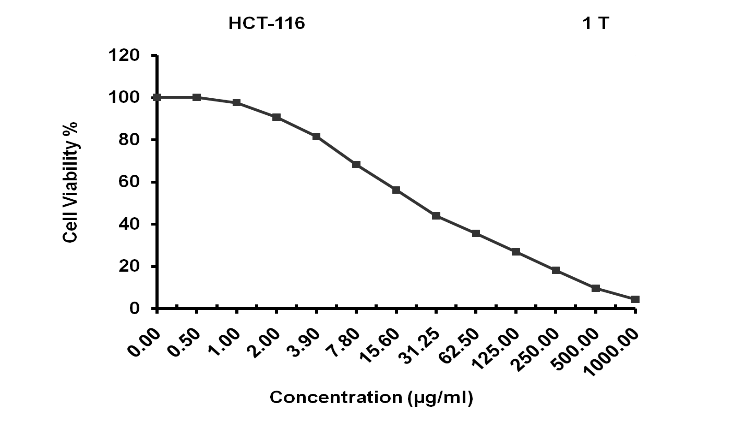


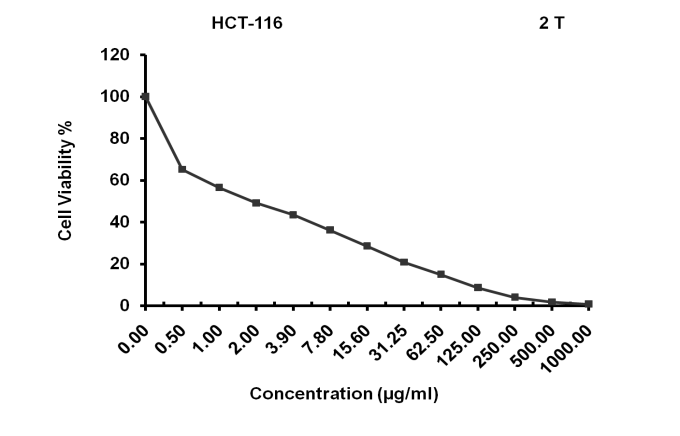


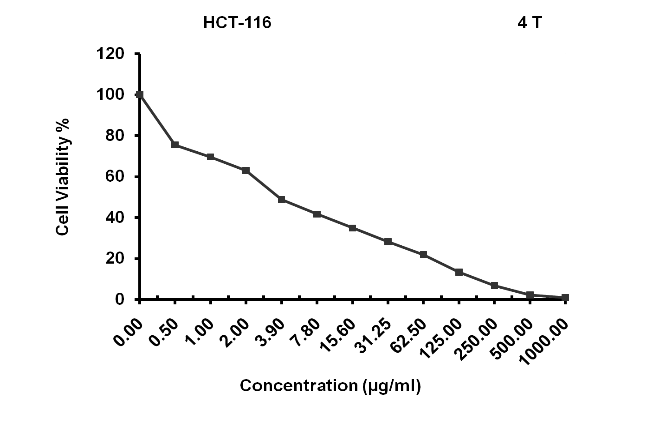

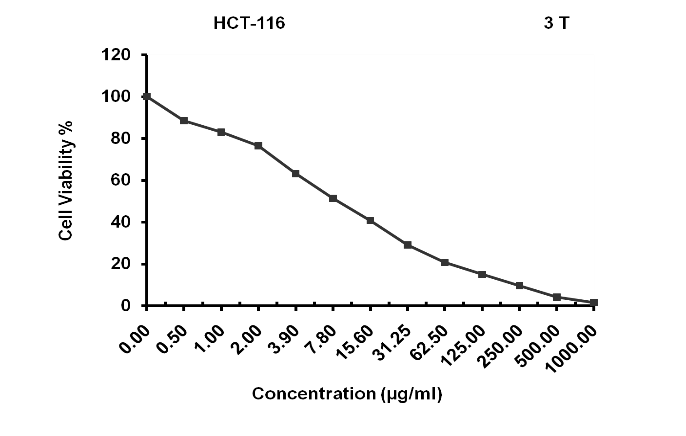


**Fig S5.** Inhibition of HCT-116 cells by the essential oil of *P. graveolens* L. 1T, 2T, 3T, and 4T represent 0, 50, 75, and 100 mM NaCl, respectively.

**Table S1.** Anticancer activity of the essential oil of *P. graveolens* L. against HepG-2 cell line

| Sample conc. (µg/ml) | 0 mM NaCl | | 50 mM NaCl | | 75 mM NaCl | | 100 mM NaCl | |
| --- | --- | --- | --- | --- | --- | --- | --- | --- |
|  | Viability % | Inhibitory % (±) S.D. | Viability % | Inhibitory % (±) S.D. | Viability % | Inhibitory % (±) S.D. | Viability % | Inhibitory % (±) S.D. |
| 1000 | 2.83 | 97.17±0.3 | 0.49 | 99.51±0.17 | 0.93 | 99.07±0.15 | 0.67 | 99.33±0.09 |
| 500 | 4.27 | 95.73±0.3 | 0.97 | 99.03±0.31 | 2.74 | 97.26±0.26 | 1.45 | 98.55±0.11 |
| 250 | 7.19 | 92.81±0.2 | 1.58 | 98.42±0.06 | 6.09 | 93.91±0.37 | 2.36 | 97.64±0.28 |
| 125 | 12.36 | 87.64±0.6 | 3.04 | 96.96±0.12 | 9.45 | 90.55±0.61 | 4.82 | 95.18±0.54 |
| 62.5 | 18.94 | 81.06±0.9 | 5.9 | 94.1±0.28 | 12.82 | 87.18±0.44 | 7.51 | 92.49±0.13 |
| 31.25 | 30.61 | 69.39±0.7 | 9.82 | 90.18±0.34 | 17.08 | 82.92±0.36 | 12.34 | 87.66±0.62 |
| 15.6 | 42.79 | 57.21±1.5 | 17.43 | 82.57±0.19 | 22.94 | 77.06±0.62 | 20.95 | 79.05±0.73 |
| 7.8 | 51.24 | 48.76±1.4 | 24.56 | 75.44±0.32 | 31.72 | 68.28±1.46 | 27.8 | 72.2±0.68 |
| 3.9 | 59.37 | 40.63±0.7 | 29.87 | 70.13±0.69 | 43.25 | 56.75±2.13 | 35.41 | 64.59±1.03 |
| 2 | 80.26 | 19.74±0.4 | 35.46 | 64.54±1.42 | 52.87 | 47.13±1.91 | 44.29 | 55.71±1.35 |
| 1 | 91.35 | 8.65±0.3 | 42.85 | 57.15±0.61 | 64.19 | 35.81±0.67 | 50.37 | 49.63±0.69 |
| 0.5 | 94.18 | 5.82±0.1 | 53.29 | 46.71±0.53 | 71.88 | 28.12±0.36 | 60.84 | 39.16±0.28 |
| 0 | 100 | 0 | 100 | 0 | 100 | 0 | 100 | 0 |

**Table S2.** Anticancer activity of the essential oil of *P. graveolens* L. against HCT116 cell line

| Sample conc. (µg/ml) | 0 mM NaCl | | 50 mM NaCl | | 75 mM NaCl | | 100 mM NaCl | |
| --- | --- | --- | --- | --- | --- | --- | --- | --- |
|  | Viability % | Inhibitory % (±) S.D. | Viability % | Inhibitory % (±) S.D. | Viability % | Inhibitory % (±) S.D. | Viability % | Inhibitory % (±) S.D. |
| 1000 | 4.21 | 95.79±0.2 | 0.65 | 99.35±0.09 | 1.45 | 98.55±0.03 | 0.79 | 99.21±0.07 |
| 500 | 9.54 | 90.46±0.3 | 1.72 | 98.28±0.06 | 4.17 | 95.83±0.15 | 2.18 | 97.82±0.06 |
| 250 | 18.06 | 81.94±0.8 | 3.98 | 96.02±0.12 | 9.58 | 90.42±0.26 | 6.75 | 93.25±0.31 |
| 125 | 26.75 | 73.25±1.6 | 8.61 | 91.39±0.07 | 14.91 | 85.09±0.33 | 13.24 | 86.76±0.62 |
| 62.5 | 35.46 | 64.54±2.1 | 14.93 | 85.07±0.11 | 20.63 | 79.37±0.75 | 21.86 | 78.14±0.48 |
| 31.25 | 43.85 | 56.15±1.7 | 20.68 | 79.32±0.16 | 28.94 | 71.06±0.42 | 28.19 | 71.81±0.37 |
| 15.6 | 56.08 | 43.92±1.8 | 28.4 | 71.6±0.42 | 40.67 | 59.33±0.79 | 34.78 | 65.22±0.26 |
| 7.8 | 68.12 | 31.88±1.4 | 36.09 | 63.91±0.35 | 51.29 | 48.71±0.35 | 41.57 | 58.43±0.39 |
| 3.9 | 81.43 | 18.57±1.5 | 43.38 | 56.62±0.14 | 63.04 | 36.96±0.96 | 48.7 | 51.3±0.28 |
| 2 | 90.58 | 9.42±0.6 | 49.07 | 50.93±0.39 | 76.38 | 23.62±0.26 | 62.83 | 37.17±0.79 |
| 1 | 97.46 | 2.54±0.3 | 56.42 | 43.58±0.84 | 82.95 | 17.05±0.11 | 69.46 | 30.54±1.28 |
| 0.5 | 100 | 0 | 65.04 | 34.96±0.68 | 88.41 | 11.59±0.37 | 75.39 | 24.61±0.75 |
| 0 | 100 | 0 | 100 | 0 | 100 | 0 | 100 | 0 |


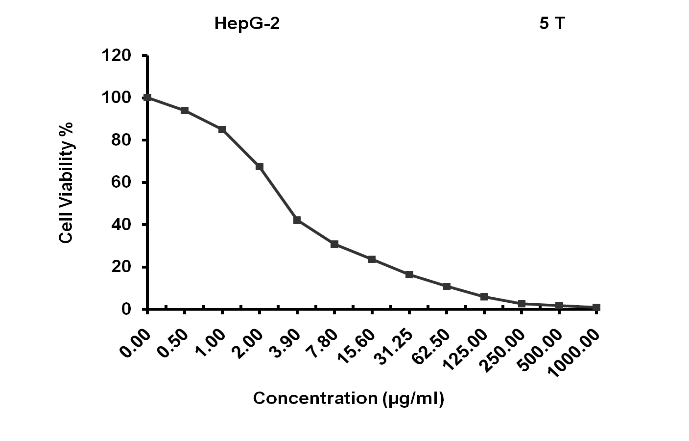

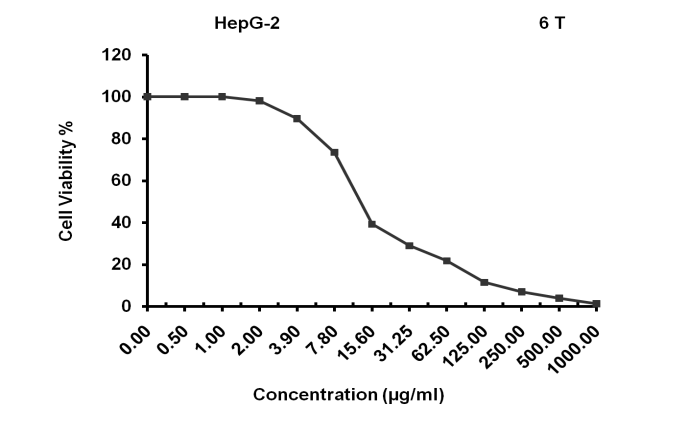


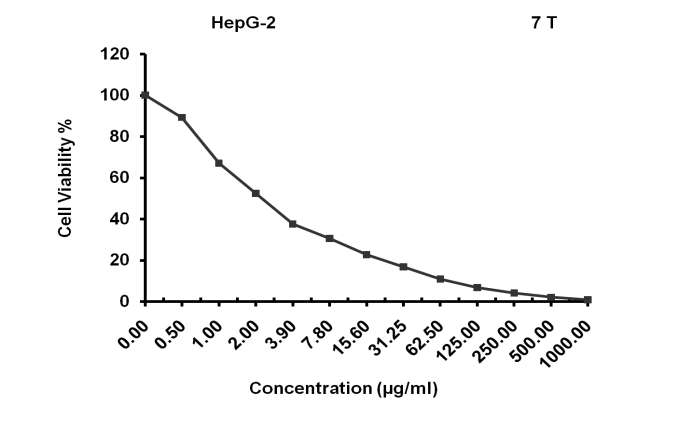

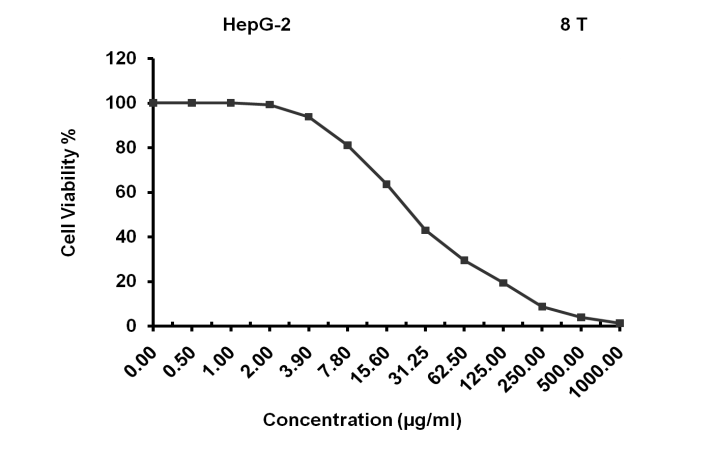
 **Fig S6.** Inhibition of HepG-2 cells by the essential oil of *M. longifolia* L. 5T, 6T, 7T, and 8T represent 0, 50, 75, and 100 mM NaCl, respectively.


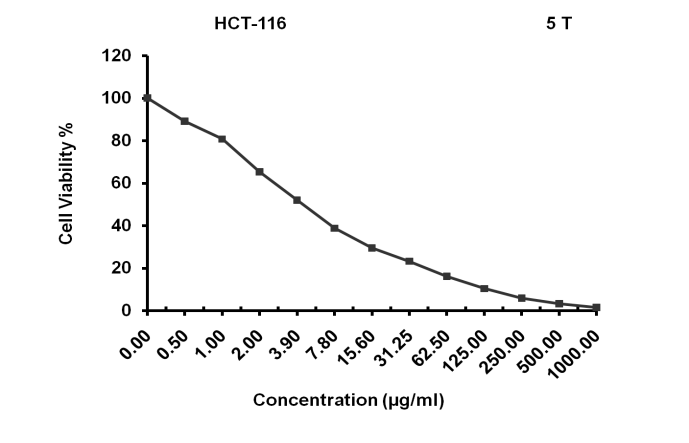

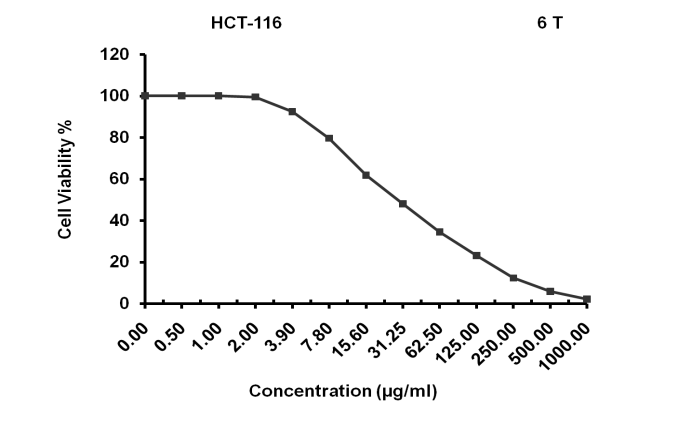


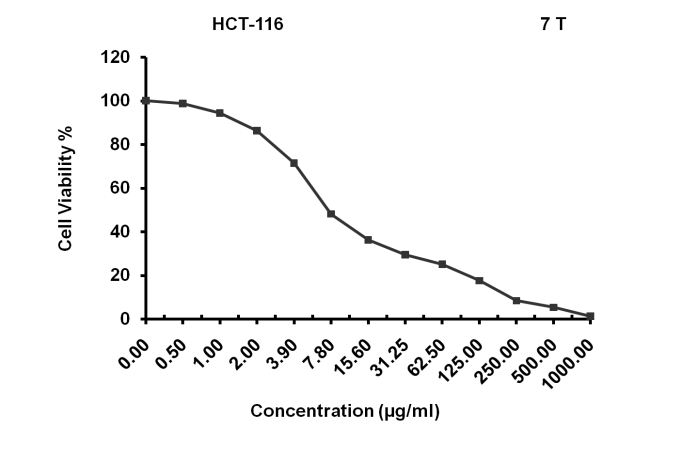

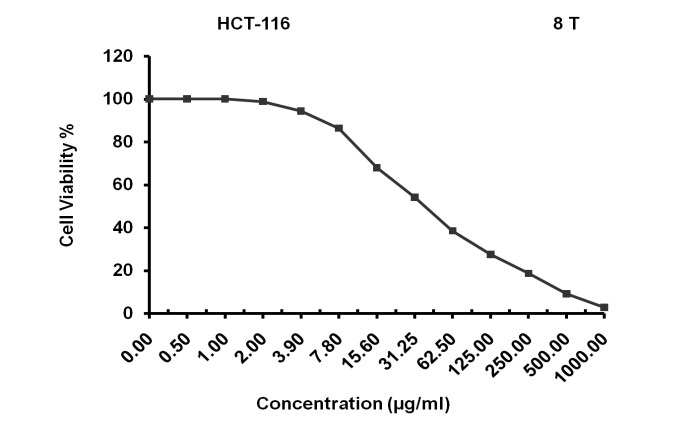


**Fig S7.** Inhibition of HCT-116 cells by the essential oil of *M. longifolia* L. 5T, 6T, 7T, and 8T represent 0, 50, 75, and 100 mM NaCl, respectively.

**Table S3.** Anticancer activity of the essential oil of *M. longifolia* L. against HepG-2 cell line

| Sample conc. (µg/ml) | 0 mM NaCl | | 50 mM NaCl | | 75 mM NaCl | | 100 mM NaCl | |
| --- | --- | --- | --- | --- | --- | --- | --- | --- |
|  | Viability % | Inhibitory % (±) S.D. | Viability % | Inhibitory % (±) S.D. | Viability % | Inhibitory % (±) S.D. | Viability % | Inhibitory % (±) S.D. |
| 1000 | 0.84 | 99.16±0.2 | 1.28 | 98.72±0.14 | 0.85 | 99.15±0.23 | 1.24 | 98.76±0.28 |
| 500 | 1.72 | 98.28±0.2 | 3.96 | 96.04±0.32 | 2.09 | 97.91±0.65 | 3.96 | 96.04±0.18 |
| 250 | 2.68 | 97.32±0.4 | 7.03 | 92.97±0.71 | 4.13 | 95.87±0.31 | 8.75 | 91.25±0.54 |
| 125 | 5.94 | 94.06±0.4 | 11.46 | 88.54±0.28 | 6.72 | 93.28±0.44 | 19.32 | 80.68±0.26 |
| 62.5 | 10.85 | 89.15±0.1 | 21.73 | 78.27±0.19 | 10.94 | 89.06±0.52 | 29.47 | 70.53±0.39 |
| 31.25 | 16.43 | 83.57±0.9 | 28.94 | 71.06±0.32 | 16.85 | 83.15±0.91 | 42.91 | 57.09±1.27 |
| 15.6 | 23.59 | 76.41±0.7 | 39.18 | 60.82±1.26 | 22.71 | 77.29±0.63 | 63.45 | 36.55±1.81 |
| 7.8 | 30.84 | 69.16±0.6 | 73.41 | 26.59±3.15 | 30.62 | 69.38±1.44 | 80.98 | 19.02±2.06 |
| 3.9 | 42.09 | 57.91±0.9 | 89.52 | 10.48±0.64 | 37.59 | 62.41±2.07 | 93.72 | 6.28±0.84 |
| 2 | 67.32 | 32.68±1.7 | 98.04 | 1.96±0.32 | 52.38 | 47.62±2.16 | 99.15 | 0.85±0.23 |
| 1 | 84.91 | 15.09±0.8 | 100 | 0 | 67.06 | 32.94±1.48 | 100 | 0 |
| 0.5 | 93.85 | 6.15±0.2 | 100 | 0 | 89.14 | 10.86±0.82 | 100 | 0 |
| 0 | 100 | 0 | 100 | 0 | 100 | 0 | 100 | 0 |

**Table S4.** Anticancer activity of the essential oil of *M. longifolia* L. against HCT116 cell line

| Sample conc. (µg/ml) | 0 mM NaCl | | 50 mM NaCl | | 75 mM NaCl | | 100 mM NaCl | |
| --- | --- | --- | --- | --- | --- | --- | --- | --- |
|  | Viability % | Inhibitory % (±) S.D. | Viability % | Inhibitory % (±) S.D. | Viability % | Inhibitory % (±) S.D. | Viability % | Inhibitory % (±) S.D. |
| 1000 | 1.43 | 98.57±0.3 | 2.16 | 97.84±0.32 | 1.16 | 98.84±0.08 | 2.76 | 97.24±0.23 |
| 500 | 3.27 | 96.73±0.1 | 5.94 | 94.06±0.18 | 5.28 | 94.72±0.14 | 9.08 | 90.92±0.46 |
| 250 | 5.81 | 94.19±0.3 | 12.35 | 87.65±0.69 | 8.34 | 91.66±0.28 | 18.64 | 81.36±0.38 |
| 125 | 10.29 | 89.71±0.5 | 23.17 | 76.83±0.75 | 17.51 | 82.49±0.35 | 27.45 | 72.55±1.79 |
| 62.5 | 16.05 | 83.95±0.3 | 34.56 | 65.44±1.22 | 25.08 | 74.92±0.46 | 38.46 | 61.54±2.08 |
| 31.25 | 23.17 | 76.83±0.3 | 48.03 | 51.97±1.95 | 29.43 | 70.57±0.61 | 54.09 | 45.91±2.13 |
| 15.6 | 29.4 | 70.6±0.2 | 61.79 | 38.21±2.13 | 36.21 | 63.79±0.93 | 67.81 | 32.19±0.67 |
| 7.8 | 38.71 | 61.29±1.4 | 79.56 | 20.44±0.82 | 48.06 | 51.94±2.12 | 86.29 | 13.71±0.35 |
| 3.9 | 51.94 | 48.06±1.7 | 92.31 | 7.69±0.57 | 71.34 | 28.66±1.28 | 94.26 | 5.74±0.12 |
| 2 | 65.23 | 34.77±1.1 | 99.42 | 0.58±0.34 | 86.2 | 13.8±0.64 | 98.74 | 1.26±0.28 |
| 1 | 80.67 | 19.33±0.3 | 100 | 0 | 94.23 | 5.77±0.15 | 100 | 0 |
| 0.5 | 89.04 | 10.96±0.6 | 100 | 0 | 98.65 | 1.35±0.37 | 100 | 0 |
| 0 | 100 | 0 | 100 | 0 | 100 | 0 | 100 | 0 |


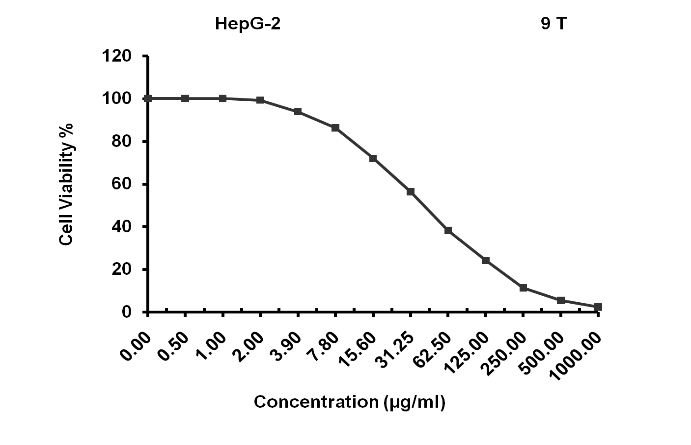

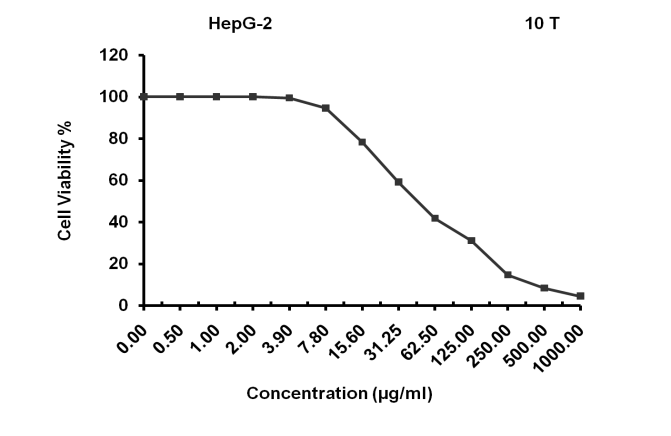


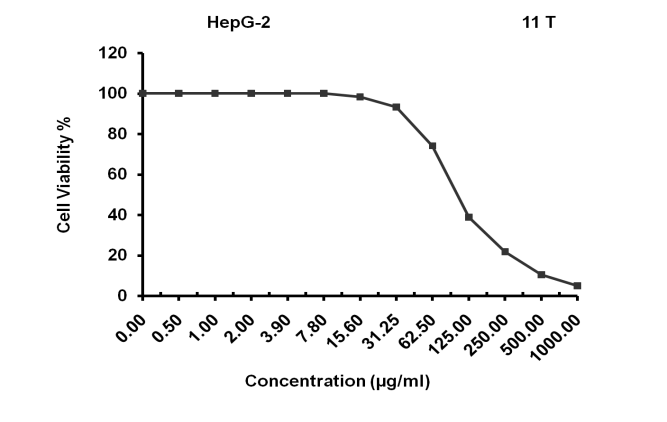

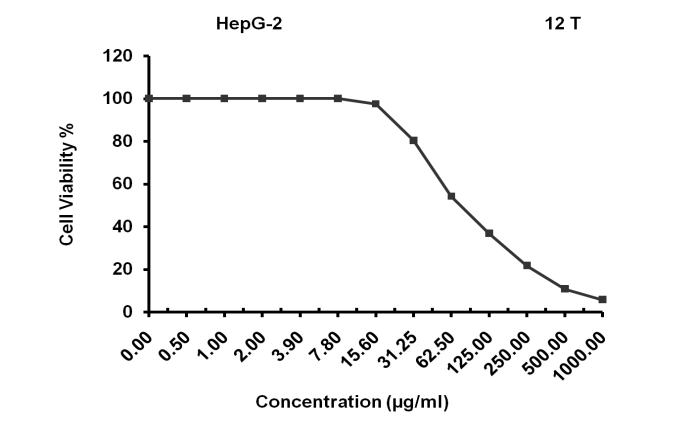


**Fig S8.** Inhibition of HepG-2 cells by the essential oil of *C. frutescens* L. 9T, 10T, 11T, and 12T represent 0, 50, 75, and 100 mM NaCl, respectively.


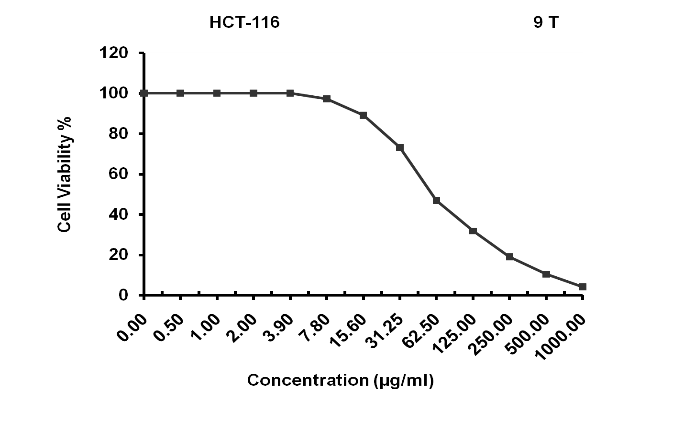

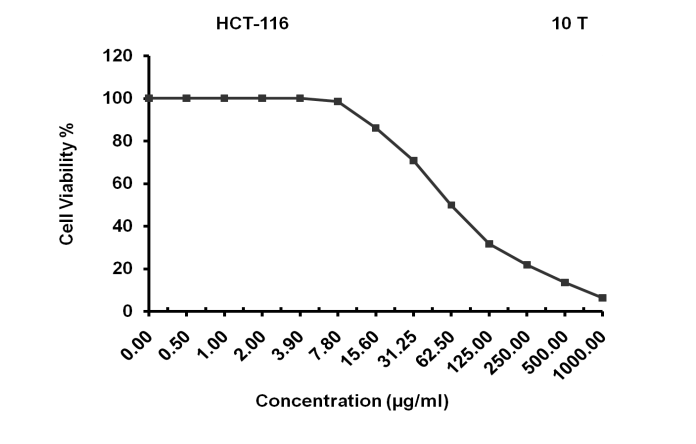


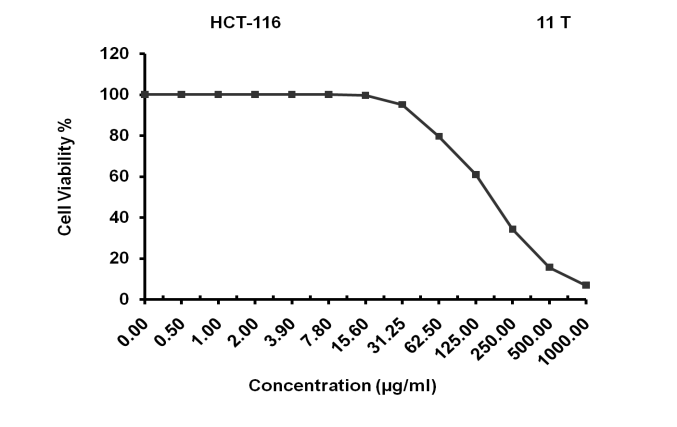

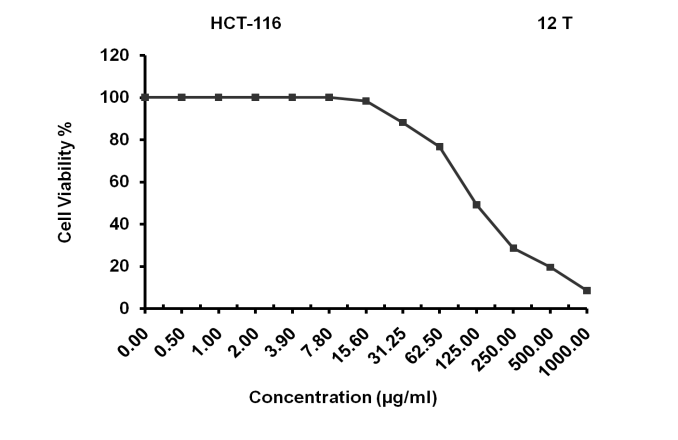


**Fig S9.** Inhibition of HCT-116 cells by the essential oil of *C. frutescens* L. 9T, 10T, 11T, and 12T represent 0, 50, 75, and 100 mM NaCl, respectively.

**Table S5**. Anticancer activity of the essential oil of *C. frutescens* L. against HepG-2 cell line

| Sample conc. (µg/ml) | 0 mM NaCl | | 50 mM NaCl | | 75 mM NaCl | | 100 mM NaCl | |
| --- | --- | --- | --- | --- | --- | --- | --- | --- |
|  | Viability % | Inhibitory % (±) S.D. | Viability % | Inhibitory % (±) S.D. | Viability % | Inhibitory % (±) S.D. | Viability % | Inhibitory %(±) S.D. |
| 1000 | 2.35 | 97.65±0.1 | 4.37 | 95.63±0.24 | 4.93 | 95.07±0.29 | 5.68 | 94.32±0.24 |
| 500 | 5.49 | 94.51±0.4 | 8.21 | 91.79±0.35 | 10.41 | 89.59±0.67 | 10.76 | 89.24±0.32 |
| 250 | 11.36 | 88.64±0.1 | 14.57 | 85.43±0.11 | 21.75 | 78.25±0.93 | 21.64 | 78.36±1.58 |
| 125 | 24.09 | 75.91±0.6 | 30.98 | 69.02±0.26 | 38.87 | 61.13±1.59 | 36.75 | 63.25±2.31 |
| 62.5 | 38.13 | 61.87±1.5 | 41.72 | 58.28±0.94 | 74.06 | 25.94±0.82 | 54.19 | 45.81±2.75 |
| 31.25 | 56.29 | 43.71±3 | 59.03 | 40.97±1.71 | 93.21 | 6.79±0.17 | 80.26 | 19.74±1.08 |
| 15.6 | 71.94 | 28.06±2.1 | 78.16 | 21.84±2.08 | 98.17 | 1.83±0.35 | 97.43 | 2.57±0.79 |
| 7.8 | 86.27 | 13.73±1.8 | 94.51 | 5.49±1.07 | 100 | 0 | 100 | 0 |
| 3.9 | 93.72 | 6.28±0.6 | 99.42 | 0.58±0.54 | 100 | 0 | 100 | 0 |
| 2 | 99.15 | 0.85±0.3 | 100 | 0 | 100 | 0 | 100 | 0 |
| 1 | 100 | 0 | 100 | 0 | 100 | 0 | 100 | 0 |
| 0.5 | 100 | 0 | 100 | 0 | 100 | 0 | 100 | 0 |
| 0 | 100 | 0 | 100 | 0 | 100 | 0 | 100 | 0 |

**Table S6.** Anticancer activity of the essential oil of *C. frutescens* L. against HCT116 cell line

| Sample conc. (µg/ml) | 0 mM NaCl | | 50 mM NaCl | | 75 mM NaCl | | 100 mM NaCl | |
| --- | --- | --- | --- | --- | --- | --- | --- | --- |
|  | Viability % | Inhibitory % (±) S.D. | Viability % | Inhibitory % (±) S.D. | Viability % | Inhibitory % (±) S.D. | Viability % | Inhibitory % (±) S.D. |
| 1000 | 4.03 | 95.97±0.2 | 6.21 | 93.79±0.35 | 6.79 | 93.21±0.57 | 8.43 | 91.57±0.75 |
| 500 | 10.27 | 89.73±0.4 | 13.49 | 86.51±0.17 | 15.62 | 84.38±0.84 | 19.54 | 80.46±0.38 |
| 250 | 18.89 | 81.11±0.5 | 21.78 | 78.22±0.26 | 34.19 | 65.81±2.39 | 28.61 | 71.39±0.77 |
| 125 | 31.74 | 68.26±0.7 | 31.62 | 68.38±0.38 | 60.82 | 39.18±1.74 | 49.08 | 50.92±0.84 |
| 62.5 | 46.86 | 53.14±2.1 | 49.81 | 50.19±1.28 | 79.54 | 20.46±0.68 | 76.63 | 23.37±1.29 |
| 31.25 | 73.12 | 26.88±1.6 | 70.65 | 29.35±0.72 | 95.07 | 4.93±0.31 | 88.02 | 11.98±0.84 |
| 15.6 | 89.05 | 10.95±0.8 | 86.03 | 13.97±0.95 | 99.54 | 0.46±0.46 | 98.16 | 1.84±0.32 |
| 7.8 | 97.13 | 2.87±0.9 | 98.46 | 1.54±0.42 | 100 | 0 | 100 | 0 |
| 3.9 | 100 | 0 | 100 | 0 | 100 | 0 | 100 | 0 |
| 2 | 100 | 0 | 100 | 0 | 100 | 0 | 100 | 0 |
| 1 | 100 | 0 | 100 | 0 | 100 | 0 | 100 | 0 |
| 0.5 | 100 | 0 | 100 | 0 | 100 | 0 | 100 | 0 |
| 0 | 100 | 0 | 100 | 0 | 100 | 0 | 100 | 0 |
